# Exploring the naturally acquired response to Pvs47 gametocyte antigen

**DOI:** 10.3389/fimmu.2024.1455454

**Published:** 2024-10-10

**Authors:** Gisele Tatiane Soares da Veiga, Rafael Amaral Donassolo, Sofia Forcellini, Julia Weber Ferraboli, Mario Antonio Kujbida Junior, Líndice Mitie Nisimura, Letícia Werzel Bassai, Rafael Luis Kessler, Mariana Serpeloni, Najara Carneiro Bittencourt, Yanka Evellyn Alves R. Salazar, Luiz Felipe Ferreira Guimarães, Jaime Louzada, Dayanne Kamylla Alves da Silva Barros, Stefanie Costa Pinto Lopes, Luzia Helena Carvalho, Tais Nóbrega de Sousa, Flora Satiko Kano, Fabio Trindade Maranhão Costa, Pryscilla Fanini Wowk, Letusa Albrecht

**Affiliations:** ^1^ Laboratório de Pesquisa em Apicomplexa, Instituto Carlos Chagas, Fundação Oswaldo Cruz (Fiocruz), Curitiba, PR, Brazil; ^2^ Grupo de Imunologia Celular e Molecular, Instituto Carlos Chagas, Fundação Oswaldo Cruz (Fiocruz), Curitiba, PR, Brazil; ^3^ Instituto de Biologia Molecular do Paraná (IBMP), Curitiba, Brazil; ^4^ Laboratório de Doenças Tropicais Prof. Dr. Luiz Jacintho da Silva, Departamento de Genética, Evolução, Microbiologia e Imunologia, Universidade de Campinas - UNICAMP, Campinas, Brazil; ^5^ Biologia Molecular e Imunologia da Malária, Instituto René Rachou, Fundação Oswaldo Cruz (Fiocruz), Belo Horizonte, Brazil; ^6^ Department of Microbiology, Tumor and Cell biology, Karolinska Institutet, Solna, Sweden; ^7^ Laboratório de Parasitologia e Monitoramento de Artrópodes Vetores na Amazônia, Centro de Ciências da Saúde, Universidade Federal de Roraima (UFRR), Boa Vista, Brazil; ^8^ Fundação de Medicina Tropical Doutor Heitor Vieira Dourado (FMT-HVD), Manaus, Brazil; ^9^ Instituto Leônidas & Maria Deane, Fundação Oswaldo Cruz (Fiocruz), Manaus, Brazil

**Keywords:** antigenicity, Pvs47, gametocyte, transmission blocking vaccine, *Plasmodium vivax*, malaria

## Abstract

Malaria represents a challenging global public health task, with *Plasmodium vivax* being the predominant parasite in Brazil and the most widely distributed species throughout the world. Developing a vaccine against *P. vivax* malaria demands innovative strategies, and targeting gametocyte antigens shows promise for blocking transmission prevention. Among these antigens, Pvs47, expressed in gametocytes, has shown remarkable efficacy in transmission blocking. However, remains underexplored in vaccine formulations. This study employed *in silico* methods to comprehensively characterize the physicochemical properties, structural attributes, epitope presence, and conservation profile of Pvs47. Additionally, we assessed its antigenicity in individuals exposed to malaria in endemic Brazilian regions. Recombinant protein expression occurred in a eukaryotic system, and antigenicity was evaluated using immunoenzymatic assays. The responses of naturally acquired IgM, total IgG, and IgG subclasses were analyzed in three groups of samples from Amazon region. Notably, all samples exhibited anti-Pvs47 IgM and IgG antibodies, with IgG3 predominating. Asymptomatic patients demonstrated stronger IgG responses and more diverse subclass responses. Anti-Pvs47 IgM and IgG responses in symptomatic individuals decrease over time. Furthermore, we observed a negative correlation between anti-Pvs47 IgM response and gametocytemia in samples of symptomatic patients, indicating a gametocyte-specific response. Additionally, negative correlation was observed among anti-Pvs47 antibody response and hematocrit levels. Furthermore, comparative analysis with widely characterized blood antigens, PvAMA1 and PvMSP1_19_, revealed that Pvs47 was equally or more recognized than both proteins. In addition, there is positive correlation between *P. vivax* blood asexual and sexual stage immune responses. In summary, our study unveils a significant prevalence of anti-Pvs47 antibodies in diverse Amazonian samples and the importance of IgM response for gametocytes depuration. These findings regarding the *in silico* characterization and antigenicity of Pvs47 provide crucial insights for potential integration into *P. vivax* vaccine formulations.

## 1 Introduction

Malaria has a significant impact on quality of life in populations residing in endemic areas. In 2022, the World Health Organization registered 249 million cases of malaria infection (1). *Plasmodium vivax* is the second most prevalent malaria parasite after *P. falciparum* in terms of the number of cases. It is the most geographically distributed parasite and is the main outside of Africa (1). In Brazil, more than 131,224 cases were reported in 2022, and approximately 84% of autochthonous cases of malaria are caused by this parasite (2). Despite its worldwide distribution, *P. vivax* has been neglected for decades (3). However, this scenario has changed in recent years since several studies reported severe cases related to this species (4–6).

*P. vivax* infections are heterogeneous and vary according to patient characteristics (7). These infections can manifest as asymptomatic, uncomplicated symptoms or progress to severe malaria. In severe cases of malaria, symptoms such as severe thrombocytopenia, severe anemia, acute respiratory syndrome, and cerebral malaria may be observed (7). Cytoadhesive features and the ability of *P. vivax* to form rosettes are suspected to be some of the factors responsible for the worsening of this disease (8, 9). Uncomplicated symptomatic malaria is the most frequently observed infection and is associated with the development of immune acquisition after exposure in endemic areas. Asymptomatic malaria is one of the greatest obstacles for parasite control since patients act as a reservoir of gametocytes without presenting symptoms, contributing to transmission (10–12). Furthermore, the development of latent hypnozoite forms in the liver establishes a reservoir for the parasite, leading to potential relapses weeks to months after infection and posing challenges to its elimination (13, 14).

The elimination of malaria is an international goal. Therefore, it is necessary to seek measures to control and block transmission. In this sense, understanding the characteristics of sexual stages, such as gametocytes, is fundamental for successful control and elimination of malaria (15). Gametocytes develop inside erythrocytes and are transmitted to female *Anopheles* during blood feeding. By ingesting these forms, the gametes merge, and the sexual cycle begins inside the mosquito midgut (16, 17). In *P. vivax* infections, gametocytes appear very quickly in the peripheral circulation and therefore allow the transmission of the parasite even before clinical symptoms and treatment (15). *P. vivax* gametocytes were found to adhere to the bone marrow (18, 19). These data support the hypothesis that these stages may migrate during infection, with the bone marrow being an important reservoir for the maturation and proliferation of parasites (20). In addition, the ability of gametocytes to form rosettes has already been demonstrated, which may favor the infection of *Anopheles* mosquitoes (21, 22). Therefore, the identification of specific *P. vivax* gametocyte proteins may assist in the development of transmission-blocking vaccines (23).

The *P. vivax* gametocyte protein 47 (Pvs47) belongs to the family of six-cysteine (6-Cys) proteins, which are fundamental for parasite development and gamete fertilization (24). The orthologous protein in *P. falciparum*, Pfs47, is located in the cytoplasm of gametocytes, and during gametogenesis, it translocates to the surface and is identified on the surface of female gametes (25). Pvs47 has also been identified in the cytoplasm of gametocytes via immunofluorescence assays (26). The presence of anti-Pvs47 hyperimmune serum significantly reduced the number of oocysts in the midgut of mosquitoes (26). Human anti-Pvs47 antibodies have also been associated with a reduced mosquito infection rate and transmission blocking ability (27). These findings indicate that Pvs47 may be a potential candidate for transmission-blocking vaccines. However, the Pvs47 protein is poorly characterized. Studies focused on analyzing physicochemical, structural, and antigenic regions are scarce. Therefore, our study used an *in silico* approach to better understand this protein, followed by the characterization of the naturally acquired response to Pvs47 in asymptomatic and symptomatic individuals of the Brazilian Amazon Region, where *P. vivax* is endemic.

## 2 Materials and methods

### 2.1 Sequence retrieval

The complete sequence of the Pvs47 protein derived from the *P. vivax* genome was retrieved in FASTA format from the National Center for Biotechnology Information^®^ (NCBI) database. The sequence (GenBank: VUZ97356.1) was used as a reference for the analyses.

### 2.2 Sequence analysis

To verify if the patterns of Pvs47 are similar to those of other 6-cys sexual proteins from *Plasmodium* species that cause malaria in humans, we selected the following sequences of each species: *P. falciparum* (ALQ43976.1), *P. knowlesi* (XP_002259881.1), *P. ovale* (SBS93970.1) and *P. malariae* (SBS97339.1). To check the conservation of the *P. vivax* antigen, we analyzed the full-length sequences of 45 sequences (433 AAs) corresponding to the Pvs47 protein deposited in the NCBI^®^. All the sequences were analyzed using AlignX in Invitrogen VectorNTI^®^ software.

### 2.3 Subcellular localization and domain analysis

Subcellular location(s) and domain analyses of the Pvs47 protein were performed using the Simple Molecular Architecture Research Tool (SMART) (28) and Prosite (29) online tools.

### 2.4 Physicochemical parameters of the Pvs47 protein

ExPASy’s ProtParam (30) was used to predict different physicochemical parameters, such as molecular weight, isoelectric point (pI), instability index, aliphatic index, and grand average of hydropathicity (GRAVY), of this protein. Solubility was checked using Protein-Sol toll (31). The possibility of glycosylation was checked by NetNglyc (32) and NetOglyc (33), phosphorylation sites were predicted by NetPhos (34), glycosylphosphatidylinositol anchor sites were predicted by NetGPI (35) and disulfide bonds predicted by the DIpro tool on the Scratch platform.

### 2.5 Antigenicity, B cell, and MHC epitope prediction

Linear B-cell epitopes in the full-length primary protein sequences were predicted by ABCpred (36). To predict regions of the antigen with high affinity for the major histocompatibility complex (MHC), we used the Immune Epitope Database (IEDB). To predict MHC-I ligands, we selected 9-10-mer peptides included in the 27 MHC-I alleles recommended by NetMHCpan EL 4.1 (37). Only sequences with percentile ranks below 0.1 and scores above 0.85 were analyzed. Similarly, the MHC-II peptides from the IEDB-recommended 2.22 method were selected to predict the epitopes of 12-18-mers using a reference set of 27 high-frequency alleles and maximal population coverage. Only regions presenting an adjusted rank of 0.2 were analyzed. The antigenicity score was evaluated using the VaxiJen (38). The conformational epitopes were generated from the modeled 3D structure, and the antibody epitope predictor Ellipro (39) was used to verify this prediction.

### 2.6 Secondary and tertiary structure prediction

The Protein Structure Analysis Workbench (PSIPRED) (40) was used for secondary structure evaluation. The tertiary structure of the protein was predicted using Iterative Threading Assembly Refinement (I-TASSER) (41). This tool predicts tridimensional structures based on the sequence-to-structure-to-function paradigm, and 3D atomic models are generated from multiple threading alignments and interactive structural assembly simulations. The improvement of the predicted model was assessed by GalaxyWeb (42). The tool can rebuild side chains, perform side-chain repacking and provides overall structure relaxation by molecular dynamics simulation. The quality of the final model was checked by a Ramachandran plot using PROCHECK (43).

### 2.7 Expression of the recombinant Pvs47 protein in the ExpiCHO eukaryotic system

The *P. vivax* Pvs47 sequence (P01 strain, PlasmoDB: PVP01_1208000), corresponding to amino acids 1 to 407, was cloned and inserted into a modified pcDNA3 plasmid (Invitrogen, Catalog Number: V79020), which contains the cytomegalovirus promoter with a signal secretion sequence and a 6xHis-tag. The protein was expressed by constitutive transfection in ExpiCHO cells (Thermo Fisher Scientific). The cells were cultured in 100 mL in Dynamis™ medium (Thermo Fisher Scientific) and maintained in a humidified incubator at 37°C with a rotation speed of 130 rpm and 8% CO_2_. Briefly, polyethylenimine (PEI) and DNA complexes were added at 3×10^6^ cells/mL. Subsequently, the transfected cells were enriched through three rounds of cell sorting by selecting cells that were positive for green fluorescent protein using flow cytometry. Fed-batch cultures of 100 mL were performed with an initial cell concentration of 6×10^6^ cells/mL. The cells were analyzed daily for density, viability, pH, and glucose levels. The pH was adjusted daily to the range of 7.2 to 7.4 using 5 M sodium bicarbonate, and when the glucose levels dropped below 3 mg/mL, Feed 2X (Thermo Fisher Scientific) was added. Conditioned media were harvested on day 8 and sterile-filtered using 0.2 µm PES filters.

### 2.8 Recombinant Pvs47 purification

The protein was purified from conditioned media through immobilized Ni Sepharose affinity chromatography utilizing a HisTrap™ Excel column (Cytiva) on the ÄKTA Pure M25 system (GE Healthcare). To equilibrate the column, 1 column volume (CV) of buffer A (30 mM Tris-HCl, 500 mM NaCl, pH 7.5) was used. Subsequently, the sample was introduced onto the column at a linear flow rate of 1 mL/min. After loading, the column was washed with 15 CV of buffer A until a stable baseline was achieved. After the washing step, protein elution occurred via a gradient ranging from 10% to 100% Buffer B (30 mM Tris-HCl, 500 mM NaCl, 500 mM imidazole, pH 7.5) (**Supplementary Figure S1A**). The eluted fractions were then subjected to anion exchange chromatography purification. These fractions were diluted in buffer A (20 mM Tris-HCL, 10% glycerol, pH=8.0) to attain a final NaCl concentration of 50 mM. Subsequently, they were loaded onto a HiTrap Q HP 1 mL column (Cytiva) prebalanced with 5% buffer B (20 mM Tris-HCL, 1 M NaCl, 10% glycerol, pH=8.0). The column underwent a wash with 10 CV and elution with 15 CV through a gradient ranging from 5% to 100% of buffer B) (**Supplementary Figure S1B**). The eluted samples were concentrated and buffer-exchanged to PBS. Approximately 3 mg of protein was obtained per liter of supernatant culture. The quality of purification was assessed by SDS-PAGE analysis using Coomassie Blue staining. Protein presence in the elution samples was confirmed via western blot analysis using a 6xHis epitope tag antibody (Invitrogen, MA121315) (**Supplementary Figure S1C**) and mass spectrometry (**Supplementary Figure S1D**). To do that, the gel containing the Pvs47 purified was subsequently destained, and the peptides were eluted for analysis using Thermo Scientific Easy-nLC liquid chromatography system coupled to an LTQ-Orbitrap XL ETD mass spectrometer (Thermo Scientific, USA). The peptides identified are showed in **Supplementary Figure S1D**.

### 2.9 *P. vivax* blood-stage recombinant proteins

The blood stage antigens PvAMA1 and PvMSP1_19_ were utilized in this study. PvAMA1 sequence was obtained from *P. vivax* isolated from Manaus (GenBank: MH049589). The PvAMA1 ectodomain fragments (nucleotides 130 to 1490) from haplotype H16 were cloned into the pGEX 4T-1 vector, expressed in *E. coli* Arctic Express (DE3), and purified using Glutathione Sepharose 4B (GE Healthcare) following the protocol detailed by Bittencourt et al., 2020 (44). To obtain PvMSP1_19_, we utilized the plasmid pET14b-MSP1_19_, which contains the 19 kDa fragment (MSP1_19_) of the *P. vivax* MSP1 protein (Belem strain, GenBank: AF435594.1), spanning amino acids 1617 to 1705, as described by Cunha et al., 2001 (45). This plasmid was generously provided by Dr. Silvia Boscardin. PvMSP1_19_ was expressed in *E. coli* NEB 5-alpha and purified using nickel affinity chromatography following the method described by Cunha et al., 2001 (45).

### 2.10 Study area and population samples

**Figure 1:**
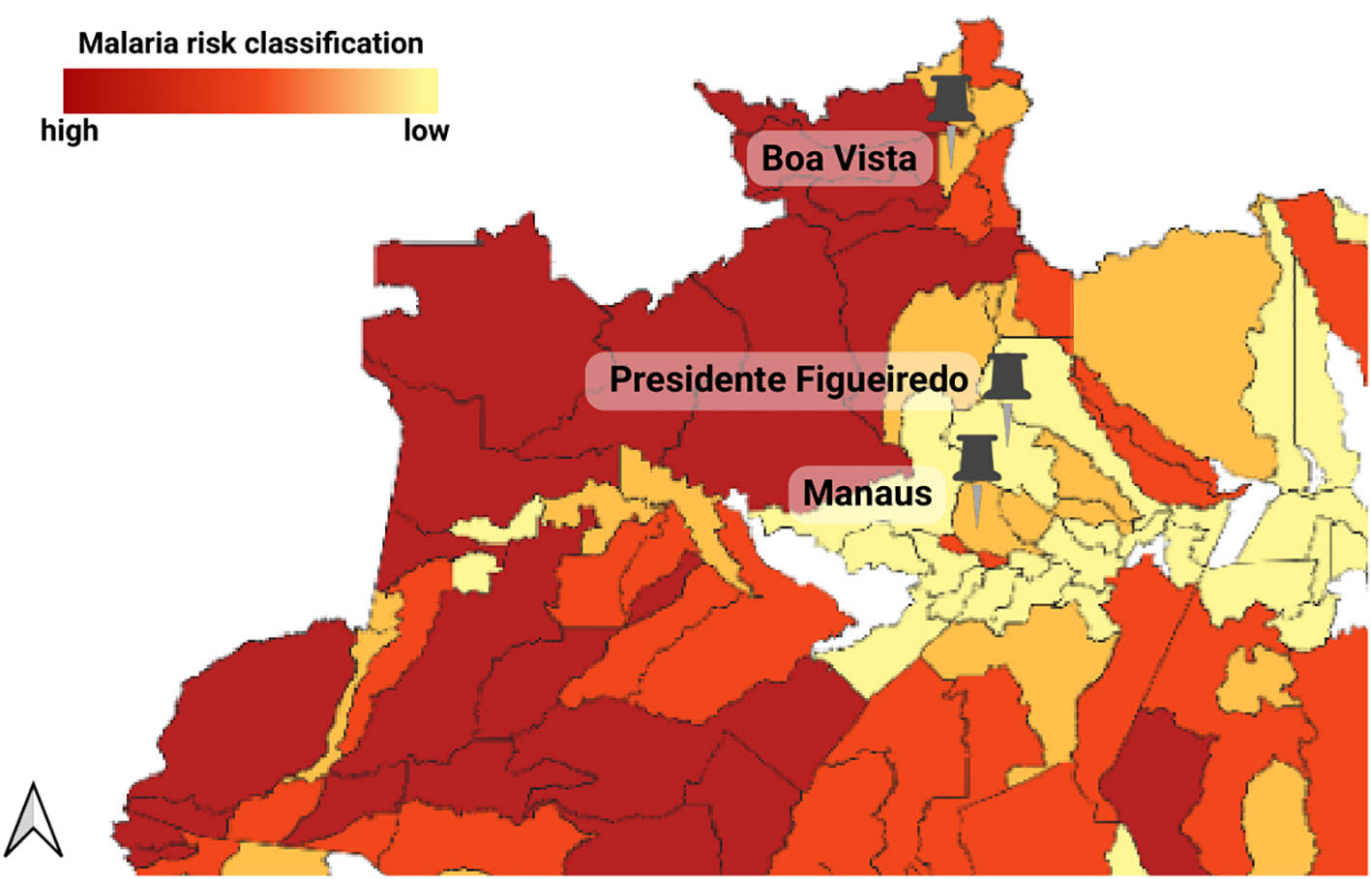
Geographical distribution of the three studied groups: symptomatic individuals from Boa Vista (Roraima), symptomatic individuals from Manaus (Amazonas), and asymptomatic individuals from Presidente Figueiredo (Amazonas). The range color indicates malaria risk levels based on the Incidence Parasite Index (IPA). The municipalities studied are classified as very low risk (IPA <1 case/1,000 inhabitants) and low risk (IPA between 1 and <10 cases/1,000 inhabitants). Adapted from Sivep-Malaria (2).

The humoral response against recombinant Pvs47, PvAMA-1 (V16) and PvMSP1_19_ were evaluated in three distinct populations samples of the Brazilian Amazon (**Table 1**): Boa Vista (Roraima), Presidente Figueiredo (Amazonas), and Manaus (Amazonas), which are all regions endemic for malaria (**Figure 1**).

**Table 1:** Population samples characteristics.

Information	Boa Vista (EC: 2751310 and 2243058)	Presidente Figueiredo (EC: 96098618.9.0000.5091)	Manaus (EC: 84250218.4.0000.0005)
**Sample number**	98	43	40
**Men (%)**	80	60	60
**Age (median, range)**	35, 17/69	39, 8/71	42.5 (20/68)
**Characteristics**	Symptomatic	Asymptomatic	Symptomatic
**Diagnostic (PCR+)**	100% *P. vivax*	56% *P. vivax* 33% *P. falciparum* 12% Coinfection (Pv/Pf)	100% *P. vivax*
**Data Assessment**	Gametocytemia (qPCR *pvs25*) Parasitemia (qPCR *pvs18S*)	Previous infection by *P. vivax*	Hematologic parameters Period of 180 days (D0, D50, D180) Gametocytemia (blood smear)
**Malaria risk classification**	Low	Very low	Low

The Boa Vista samples (Ethics Committee: 2751310 and 2243058) consisted of symptomatic patients (n=98) with acute *P. vivax* monoinfection who had experienced fever within the last 48 hours, comprising 80% men with age ranging from 17 to 69 years. Data on parasitemia (estimated by qPCR of the 18S rRNA gene in copies/μL) and gametocyte count (estimated by qPCR of the *pvs25* gene in copies/μL), as described above, were collected for these patients.

The Presidente Figueiredo samples (Ethics Committee: 96098618.9.0000.5091) comprised *P. vivax* (n=24), *P. falciparum* (n=14) or coinfected (n=5) asymptomatic individuals, microscopy negative and diagnosed by qPCR (46), from Rio Pardo settlement. Screening was performed on a sample of adults from households who expressed interest in participating and reported no symptoms of malaria at the time of enrollment, with 60% of participants being men and a median age of 39 years. The patients have an average of 8 years of residence in Rio Pardo and 33 years in the Amazon region. All individuals were negative by microscopy diagnosis on thick blood smears and positive by real time PCR (46). While all individuals had a history of prior *P. vivax* infection, at the time of PCR diagnosis, 56% tested positive for *P. vivax*, 33% for *P. falciparum*, and 12% presented coinfection with both *P. vivax* and *P. falciparum*. On average, they report 12 previous episodes of malaria (ranging from 1 to 48 previous infections).

The Manaus samples (Ethics Committee: 84250218.4.0000.0005) consisted of symptomatic patients (n=40) monitored over a period of 180 days, with ages ranging from 20 to 68 years. They were diagnosed with malaria by blood smear at the Tropical Medicine Foundation Hospital. These patients were invited to participate in the study after confirmation of *P. vivax* infection on day 0 (D0). All patients were treated, after blood sampling at D0, according to the Brazilian guidelines. Initially, 40 individuals participated (D0), with 30 returning on day 50 (D50) and only 12 on day 180 (D180), with some experiencing recurrence malaria between D0 and D50 (3/30) and between D50 and D180 (6/12). *P. vivax* infection was confirmed through microscopy and quantitative PCR (qPCR). Approximately 57.5% of participants reported prior episodes of malaria to sampling. Following blood collection, a complete blood count was performed using a Sysmex KX21N analyzer (Sysmex Corporation-Roche, Japan).

### 2.11 Parasite quantification by qRT-PCR

Blood samples from individuals in Boa Vista were quantified for total parasitemia (*18S* rRNA transcripts) and gametocytemia (*pvs25* transcripts) using quantitative PCR methods detailed by Salazar et al. (47). In summary, parasite RNA was extracted from the blood samples and subjected to quantitative reverse-transcription PCR (qRT-PCR) to generate complementary DNA (cDNA). The primer sequences specific for amplifying the *18S* rRNA and *pvs25* targets previously documented by Wampfler and colleagues (48) were applied for *P. vivax* quantification.

### 2.12 Detection of naturally acquired antibodies to recombinant antigens

Naturally acquired antibodies against recombinant Pvs47, PvAMA-1 and PvMSP1_19_, including IgM, IgG, and IgG subclasses (IgG1, IgG2, IgG3 and IgG4) specific for Pvs47, were measured in plasma samples using an indirect enzyme-linked immunosorbent assay (ELISA). High-protein binding 96-well ELISA plates were coated with 50 μL of recombinant protein at concentration of 5 μg/mL in 0.05 M carbonate-bicarbonate buffer, pH 9.6, overnight at 4°C. Following this, 200 µL of blocking solution (PBS-Tween-20 containing 5% of nonfat milk) was added, and the plates were incubated at 37°C for 1 hour. Subsequently, the wells were washed three times with 200 µL of PBS-T (PBS containing 0.05% of Tween-20) prior to sample addition. Plasma samples diluted 1:100 in PBS-T were then added to each well and incubated for 1 hour at room temperature. For the detection of bound antibodies, the samples were incubated with a 2 µg/ml dilution of peroxidase-conjugated goat anti-human IgG or IgM (Sigma Aldrich). For enzymatic reaction, a 100 µL solution of 3,3’,5,5’-Tetramethylbenzidine/H_2_O_2_ was added to the wells and incubated at room temperature for 5 minutes in the dark. The reaction was stopped by adding 100 µL of HCl 1M. Optical density (OD) was measured at 450 nm using a Synergy H1M2F plate reader. The detection of IgG subclasses was performed with specific secondary monoclonal mouse anti-human antibodies (Abcam) for IgG1 (ab99774), IgG2 (ab99779), IgG3 (ab99829), and IgG4 (ab99823) diluted to 2 µg/ml. The cutoff value was calculated as the mean plus three or ten (for IgM) standard deviations of the negative control (n=20, healthy individuals). The higher SD cutoff for IgM was chosen to ensure specificity due to the typically lower concentration and variability of IgM antibodies compared to IgG. Reactivity indices (RIs) were obtained by the ratio of the absorbance values of each sample to the cutoff value. The prevalence of antibodies against the antigens was considered positive if the RIs were greater than 1.0. Correlation analyses with gametocytemia were conducted solely on positive samples from Boa Vista and Manaus. Additionally, correlation analyses with blood counts were exclusively performed on samples from Manaus, encompassing both positive and negative cases. The IgM response was categorized as low when the Reactivity Index (RI) was between 1.0 and 1.5 and as high when the RI exceeded 1.5. This represents a value above the median of RI observed in samples with positive reactivity for IgM antibodies. In the **Supplementary Materials**, the data were also presented as arbitrary units of optical density for Pvs47 and MSP1_19_, with optical density values of the media for each sample.

### 2.13 Statistical analysis

All analyses were performed using the GraphPad Prism 8.2.1 program (GraphPad Inc., USA). The Shapiro−Wilk test was used to determine the normality of the data. For the analysis of continuous variables with an abnormal distribution and unpaired data, the differences between two groups were evaluated with the Mann−Whitney test, and for more than two groups, differences were evaluated with the Kruskal−Wallis test. For unpaired data with a normal distribution, ANOVA was used. In the analysis of paired data, the t test was used for samples with a normal distribution, and the Wilcoxon test was used for samples with an abnormal distribution. Correlations were determined by the Spearman R test, p-values was corrected using the Holm method. The data were considered significant when the p-value was lower than 0.05.

## 3 Results

### 3.1 *In silico* properties of Pvs47

Pvs47 consists of 433 amino acid (AA) residues that form a 49 kDa protein. The theoretical isoelectric point (pI) of this protein is 6.44, and in this protein, more negatively charged AA residues (59 residues) were found than positively charged AA residues (55 residues). The instability index was 33.34, which indicated that the protein was stable. The aliphatic index of a protein is defined as the relative volume occupied by its aliphatic side chains (alanine, valine, isoleucine, and leucine), which was computed to be 87.07. A high aliphatic index (>80) indicates that a protein is thermo-stable over a wide temperature range. The GRAVY value was negative (-0.321), indicating that this protein is nonpolar. Finally, the solubility of the protein (without the transmembrane region) was 0.427, indicating insolubility The number and percentage of Pvs47 amino acid composition is depicted in **Supplementary Table S1**.

**Figure 2:**
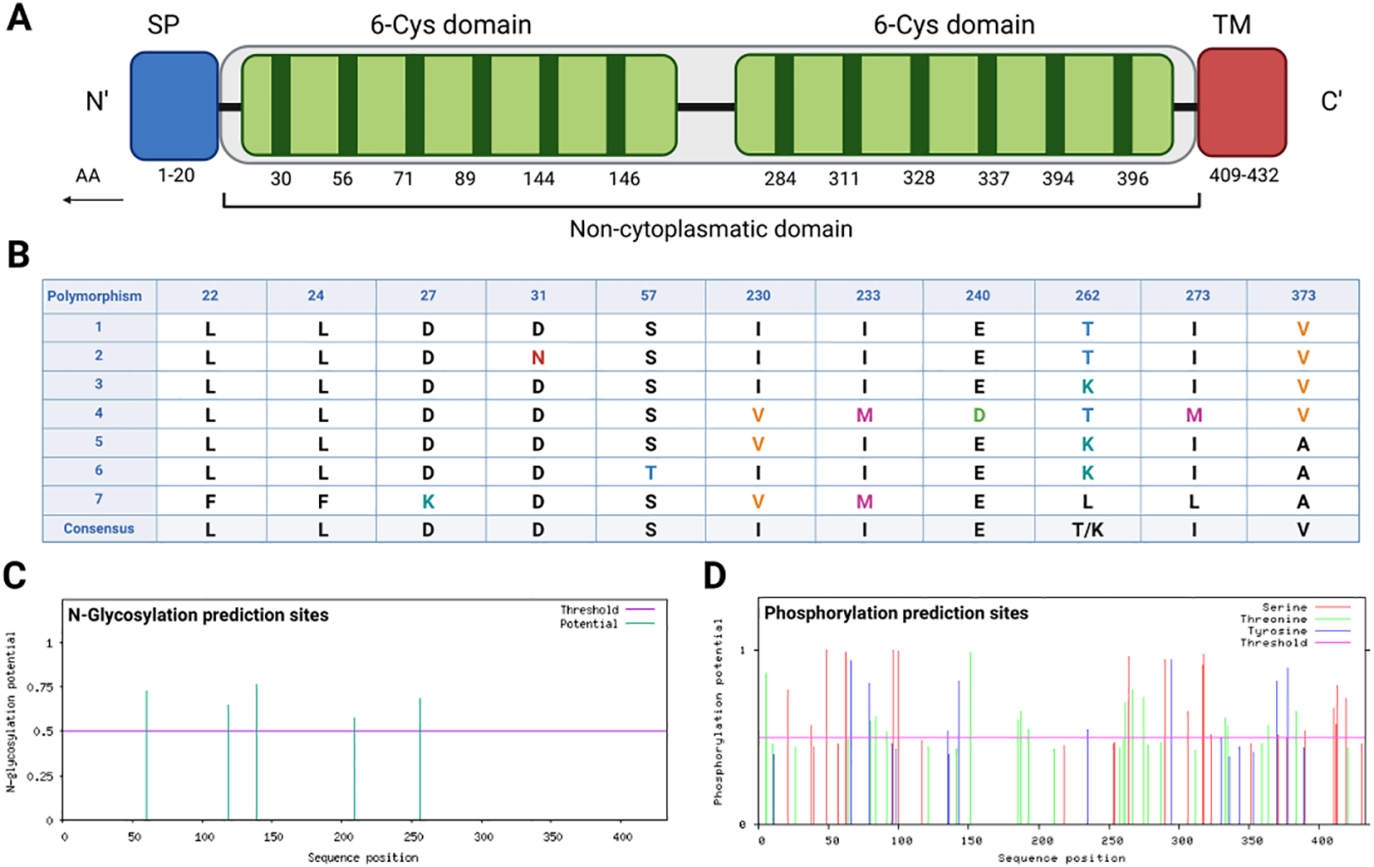
Pvs47 amino acid sequence analysis. **(A)** Predicted domains along the Pvs47 sequence. The protein contains a signal peptide (SP), two 6-cysteine domains (6-Cys domain), and a transmembrane region (TM). The residue numbers are annotated below the domain blocks in the figure. **(B)** Polymorphisms observed among 46 Pvs47 sequences. Sequences deposited in GenBank were analyzed, with seven chosen to represent the diversity. Eleven polymorphisms were identified, and the mutations with corresponding amino acid positions are illustrated in the figure. **(C)** The N-glycosylation sites predicted along the Pvs47 sequence. The results were generated by the NetNglyc server. **(D)** The phosphorylation sites predicted along the Pvs47 sequence. The results were generated by the NetPhos server.

The Pvs47 protein has two 6-cys domains. The first domain is located between regions 26-175 (30, 56, 71, 89, 144 and 146 residues), and the second 6-Cys domain is located between regions 280-414 (284, 294, 311, 328, 337 and 396 residues). Furthermore, the antigen includes a signal peptide at the N-terminus (1-20 AA), a transmembrane domain (409-431 AA) and a noncytoplasmatic site (21-413 AA) (**Figure 2A**).

The amino acid sequences of five different 6-cys family antigens from *Plasmodium* species that cause human malaria were retrieved from the NCBI database in FASTA format. According to multiple sequence alignment results, low identity (approximately 50%) was obtained among sequences from different species. The species with the highest percent identity were *P. knowlesi* (61.42%), *P. malariae* (50.36%), *P. ovale* (46.98%) and *P. falciparum* (42.15%).

No conservation pattern was found in the sequences except for the 6-cys regions, and the conserved regions were heterogeneously distributed. The conservation rate analyses between the Pvs47 sequences deposited in the databank revealed 11 polymorphisms (22, 24, 27, 31, 57, 230, 233, 240, 262, 273 and 373 AA) in the sequences and a percentage of 97.5% identity (**Figure 2B**).

The posttranslational modification pattern of Pvs47 was also examined *in silico*. The potential for glycosylation was evaluated for both N-linked and O-linked glycosylation sites. Glycosylation analysis revealed a diverse distribution of 5 asparagine-glycosylated residues within the region spanning amino acids 60 to 256 (**Figure 2C**). Concerning O-glycosylation, a lone site was predicted for glycosylation, specifically corresponding to a serine residue at position 96 (S96). Additionally, phosphorylation analysis revealed a varied distribution of phosphorylated residues, encompassing serine, threonine, or tyrosine residues, throughout the entire amino acid sequence (**Figure 2D**). Moreover, the prediction for GPI-Anchored indicated a single site, aligning with a serine residue at position 411 (S411). The prediction of disulfide bonds in the Pvs47 protein indicates the presence of six binding sites between cysteines, located at the following positions: 18-30, 71-89, 227-257, 284-311, 328-337 and 396-433. These disulfide bonds can influence the protein’s conformation.

### 3.2 Prediction of B-cell and T-cell epitopes

**Figure 3:**
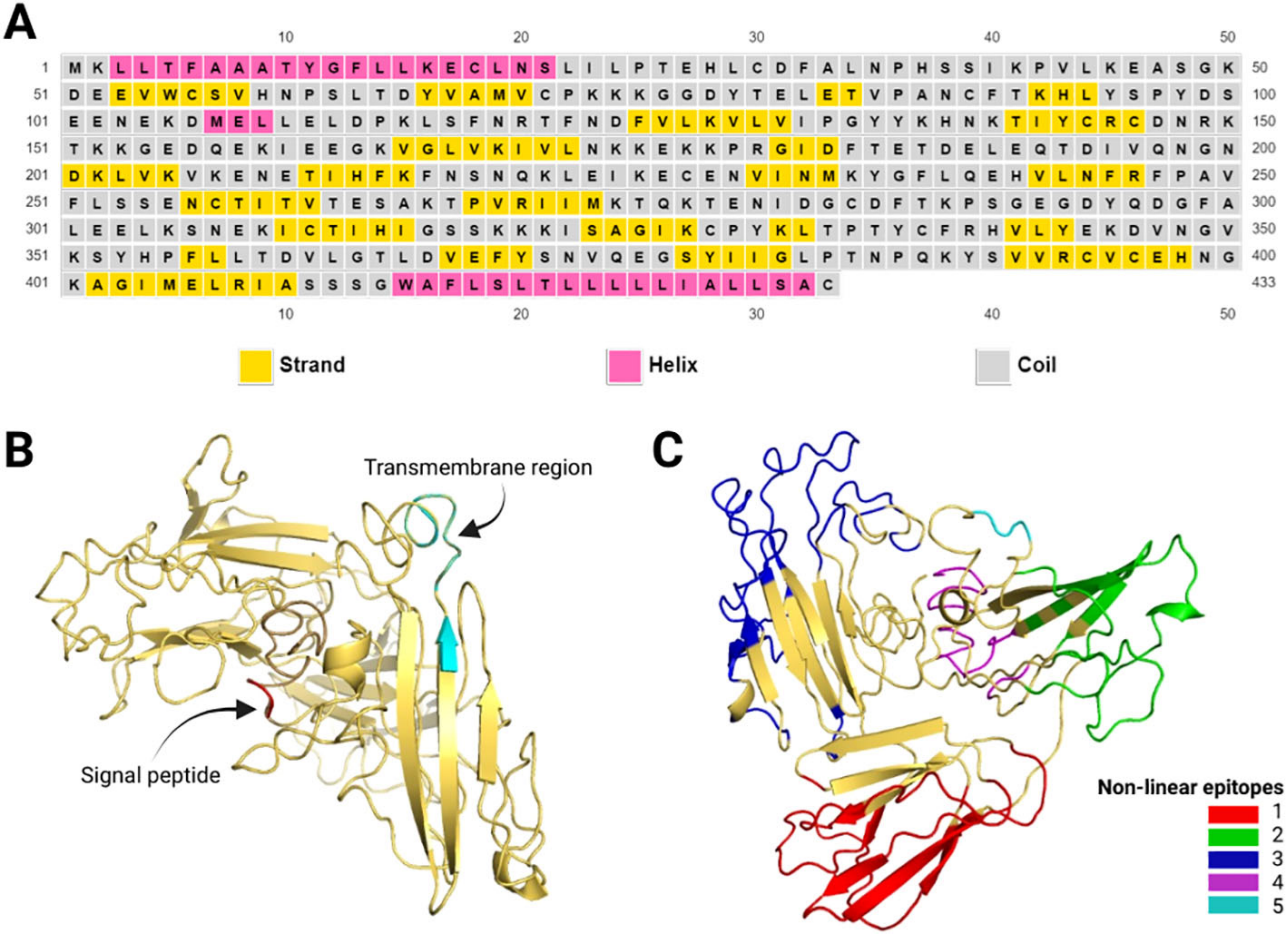
Pvs47 predicted structure. **(A)** Secondary structure of the proposed antigen according to the PSIPRED tool. The helical structures are represented in pink, the strands in yellow, and the coils in gray. **(B)** Pvs47 tridimensional structure after modeling, refinement, and validation. The signal peptide is represented in red and the transmembrane region in cyan. **(C)** Structural position of the 5 non-linear B-cell epitopes described in the **Supplementary Table S5**. Each of the colors represents a different epitope.

The prediction of epitopes showed a heterogeneous distribution for linear B and T-CD8^+^ cells in the sequence. However, for T-CD4^+^ cells, the epitopes are located between a leucine-rich region covering amino acids 408 to 421, presumably because this region comprises the transmembrane region (414–432). The tables show the epitopes with the highest scores (**Supplementary Tables S2**–**S4**). After modeling the 3D structure, it was possible to predict nonlinear B-cell epitopes (**Supplementary Table S5**, **Figure 3C**).

### 3.3 Structural characterization and 3D modeling of Pvs47

The secondary structure of the Pvs47 protein was estimated using the PSIRED tool. Random coils are predominant in proteins (65%), followed by extended strands (26%) and alpha helices (9%), which are less prevalent. A graphical representation of the secondary structure is shown in **Figure 3A**. Since there is no significant similarity with existing resolved structures, the 3D structure of this antigen cannot be predicted by homology modeling. We predicted the 3D structure using template-based modeling (TBM) and ab initio modeling with the I-Tasset tool (**Figure 3B**). The C-score of the best model was acceptable at -0.97. The C-score ranges from -5 to 2, where higher values indicate better-quality models. Furthermore, we submitted the 3D structure to improve the model quality. To validate the generated model, we used Ramachandran plot analysis. After refining the model quality, 75.6% of the residues were in the most favored regions, 18% of the residues were in additional allowed regions, 2.3% of the residues were in generously allowed regions, and 4.1% of the residues were in disallowed regions. The structural positions of the 5 predicted epitopes (**Supplementary Table S5**) are depicted in **Figure 3C**. The model is available in ModelArchive (accession code: ma-5v61z).

### 3.4 Anti-Pvs47 antibodies were detected in different samples population exposed to *P. vivax*

**Figure 4:**
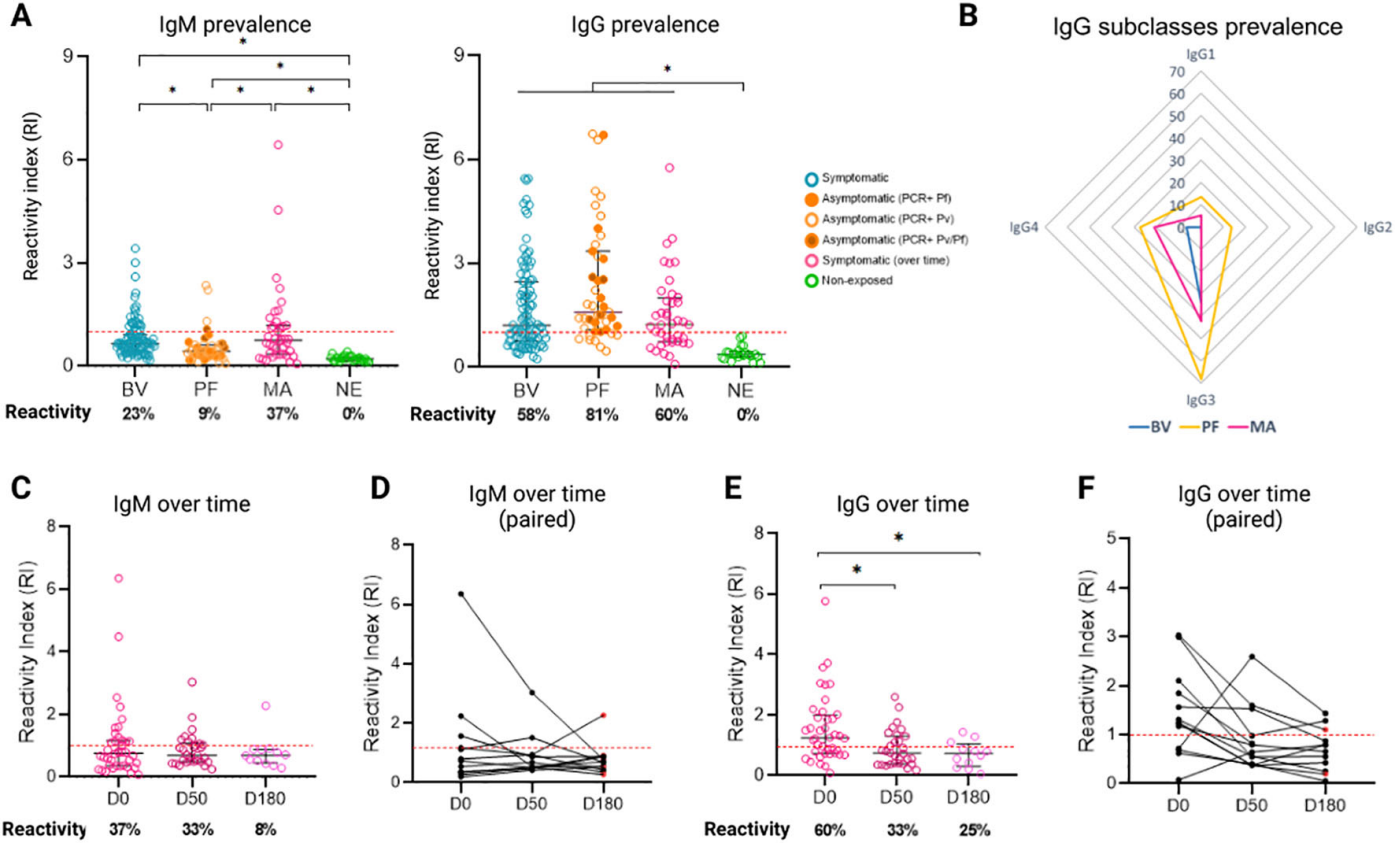
Prevalence of anti-Pvs47 antibodies in three different samples from the Brazilian Amazon. **(A)** Reactivity index (RI) for IgM and IgG among symptomatic individuals from Boa Vista (BV), asymptomatic individuals from Presidente Figueiredo (PF), Manaus (MA), and nonexposed (NE) individuals. Reactivity is represented as a percentage below the chart. **(B)** Radar charts representing the reactivity of the IgG1, IgG2, IgG3, and IgG4 subclasses in Boa Vista (blue), Manaus (pink) and Presidente Figueiredo (yellow). **(C)** IgM reactivity index and positivity percentage at D0, D50, and D180 in symptomatic individuals from Manaus. **(D)** The IgM reactivity index demonstrating the trend of reduction for each individual from Manaus. **(E)** IgG reactivity index and positivity percentage for D0, D50, and D180 from Manaus. **(F)** The IgG reactivity index demonstrating the trend of reduction for each individual from Manaus. The red points **(D, F)** at D50 (1/12) and D180 (6/12) indicate patients who tested positive for malaria again at the time of collection. The RI was calculated from the OD of individual samples divided by the cutoff, with samples with an RI > 1 (indicated by the red dotted line) considered positive. Each circle represents the response of an individual. The data from independent experiments are presented as median with interquartile range. p values were determined using the Kruskal−Wallis test, followed by Dunnett’s multiple comparison test (* p < 0.05).

The prevalence of IgM antibodies and IgG was evaluated in three groups of samples from patients with vivax malaria. It was observed that symptomatic individuals have a higher prevalence of IgM antibodies than asymptomatic individuals (**Figure 4A**, **Supplementary Figure S2**). In the Boa Vista sample (n=98), the prevalence was 23%, with a reactivity index (RI) ranging from 1 to 4, while in the Manaus samples (n=40, day 0), the prevalence was 37%, with a greater RI, ranging from 1 to 6. Instead, the sample from Presidente Figueiredo (n=43) showed a prevalence of 9%, with a low amplitude of response, RI ranging from 1 to 2. In addition, there was a significant difference (p<0.05) between the reactivity index of the infected groups and the control group, as well as between the symptomatic and asymptomatic groups.

Regarding the prevalence of IgG, it is noted that asymptomatic individuals have a higher prevalence of antibodies compared to symptomatic individuals, although there was no statistically significant difference between the groups. In the Boa Vista samples, the prevalence was 58%, while in the Manaus samples (day 0), the prevalence was 60%. On the other hand, in the samples of Presidente Figueiredo (Rio Pardo), the prevalence was 81%. It is noteworthy that in Presidente Figueiredo, some individuals had infections by *P. falciparum* (14/43), *P. vivax* (23/43) or co-infection with both species (6/43). However, all individuals were diagnosed at some point in their lives with *P. vivax* and no significant differences were observed between the three groups, so they were analyzed together. In addition, there was a significant difference (p<0.05) between the reactivity rates of the infected groups and the control group.

To assess which subclass predominates in the anti-Pvs47 response, individuals with RI>1 to anti-Pvs47 IgG were evaluated for their reactivity to IgG1, IgG2, IgG3, and IgG4 subclasses (**Figure 4B**). Symptomatic individuals from Boa Vista (n=61) exhibited 34% reactivity to IgG3 and 6.5% to IgG4 (**Figure 4B**). On the other hand, asymptomatic individuals in Presidente Figueiredo (n=22) showed 13% reactivity to IgG1, 13% to IgG2, 68% to IgG3, and 27% to IgG4 (**Figure 4B**). In the Manaus samples on D0 (n=40), the predominant responses were IgG1 (5%), IgG3 (42%), and IgG4 (21%), with no detectable IgG2 responses in any individual (**Figure 4B**). These results indicate that the prevailing subclass response against Pvs47 is IgG3 antibodies.

### 3.5 The naturally acquired response against Pvs47 over time in the Manaus samples

To assess the longevity of naturally acquired antibodies against Pvs47, the response of IgM and IgG and their subclasses was evaluated at three different time points: at the time of infection diagnosis (D0), 50 days after infection diagnosis (D50), and 180 days after infection diagnosis (D180). IgM and IgG antibodies against Pvs47 were detected throughout the analyzed period (**Figures 4C-F**). The IgM response decreased over time, starting at 37% (15/40) reactivity at D0, decreasing to 33% (10/30) at D50, and remaining at 8% (1/12) at D180 (**Figures 4C, D**). However, despite this trend, no statistically significant difference was noted (p>0.05). Moreover, the IgG response also exhibited a reduction, beginning at 60% (24/40) at D0, decreasing to 33% (10/30) at D50, and remaining at 25% (3/12) at D180 (**Figures 4E, F**). In this case, a significant difference was observed between D0 and D50 (p=0.0142) and D0 and D180 (p=0.0189). On day 50 and day 180, some individuals experienced malaria recurrence (3/30 and 6/12, respectively), as indicated by positive thick smears. Nevertheless, there was no difference in the antibody response observed between these patients and the other patients.

### 3.6 Anti-Pvs47 IgM antibody response is correlated with gametocytemia levels, but not that of IgG

**Figure 5:**
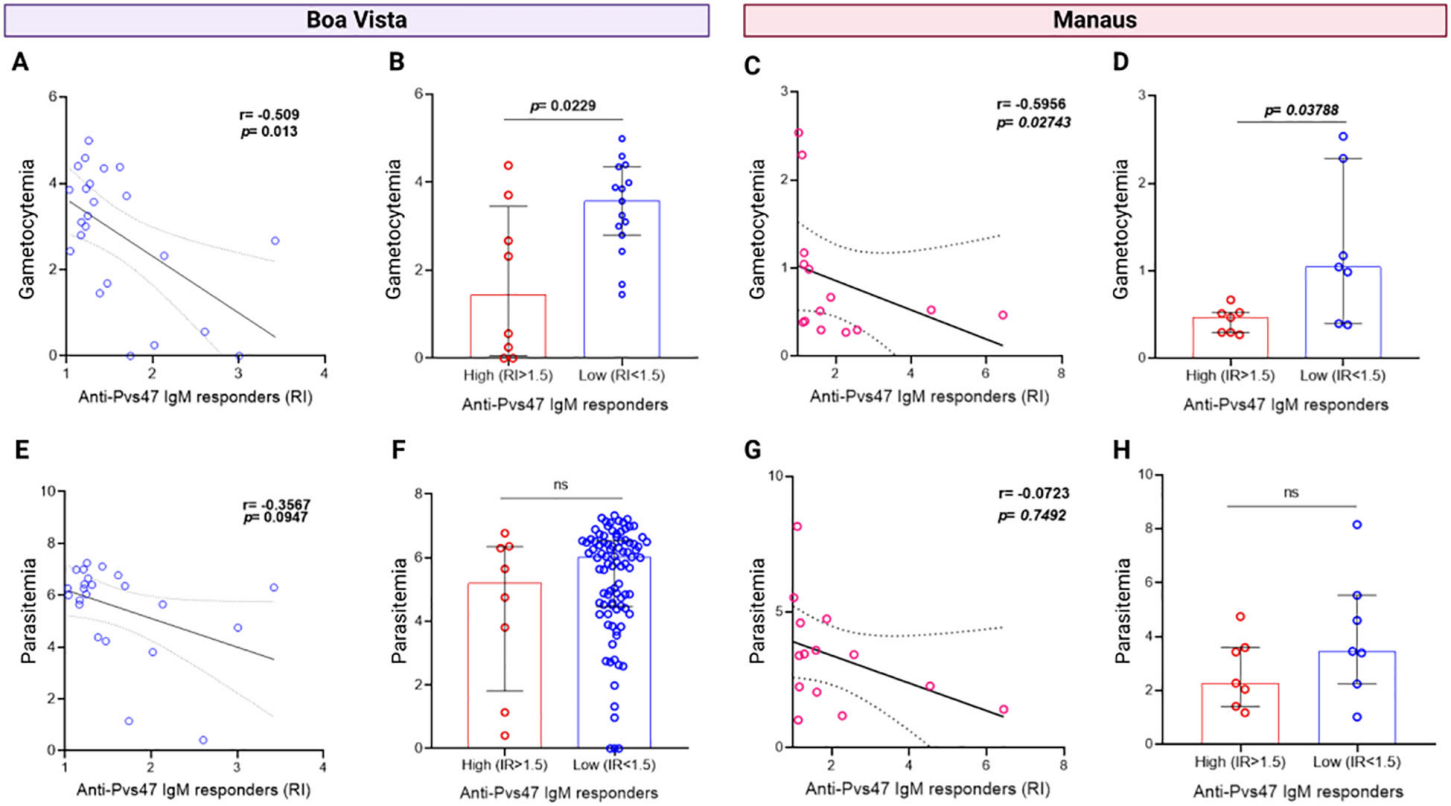
Relationship between anti-Pvs47 IgM antibody response and gametocytemia or parasitaemia levels. **(A)** Correlation between the reactivity index (RI) of the anti-Pvs47 IgM response and the levels of gametocytemia in samples from Boa Vista. **(B)** Comparison of gametocytemia levels between groups of high (RI>1.5) and low response (RI<1.5) to anti-Pvs47 IgM in samples from Boa Vista. **(C)** Correlation between the reactivity index (RI) of the anti-Pvs47 IgM response and the levels of gametocytemia in samples from Manaus at D0. **(D)** Comparison of gametocytemia levels between groups of high (RI>1.5) and low response (RI<1.5) to anti-Pvs47 IgM in samples from Manaus at D0. **(E)** Correlation between the reactivity index (RI) of the anti-Pvs47 IgM response and the levels of parasitaemia in samples from Boa Vista. **(F)** Comparison of parasitaemia levels between groups of high (RI>1.5) and low response (RI<1.5) to anti-Pvs47 IgM in samples from Boa Vista. **(G)** Correlation between the reactivity index (RI) of the anti-Pvs47 IgM response and the levels of parasitaemia in samples from Manaus at D0. **(H)** Comparison of parasitaemia levels between groups of high (RI>1.5) and low response (RI<1.5) to anti-Pvs47 IgM in samples from Manaus at D0. Circles indicate the response of each individual. Data from independent experiments are shown as median with interquartile range. The difference between the groups was evaluated by the Mann−Whitney test, and the correlation by the Spearman test was considered significant when p<0.05. In the correlation analyses, the linear regression line of the data with a 95% confidence interval is represented.

To verify the specificity of the anti-Pvs47 response towards gametocytes, we conducted correlation analyses between parasitemia and gametocytemia with IgM or IgG reactivity indices in symptomatic patient samples. We identified a negative correlation between the IgM response and gametocyte levels in Boa Vista (R= -0.509, p= 0.013) (**Figure 5A**) and Manaus (R= -0.5956, p= 0.02743) (**Figure 5C**). Moreover, upon comparing gametocytemia between patients with high and low IgM responses, we observed that those with higher responses harbored fewer gametocytes for Boa Vista (p= 0.0229) (**Figure 5B**) and Manaus (p= 0.02743) (**Figure 5D**). Intriguingly, there was no correlation (**Figures 5E, G**) or significant differences between IgM antibody responses and total parasitemia in both samples (**Figures 5F, H**). Furthermore, no correlation was observed between anti-Pvs47 IgG responses and gametocytemia or parasitemia (**Supplementary Figure S3**) and no correlation was observed for IgM or IgG and anti-PvAMA1 and anti-PvMSP1_19_ responses (**Supplementary Figure S4**).

### 3.7 The anti-Pvs47 IgM antibody responses negatively correlate with the hematocrit percentage

**Figure 6:**
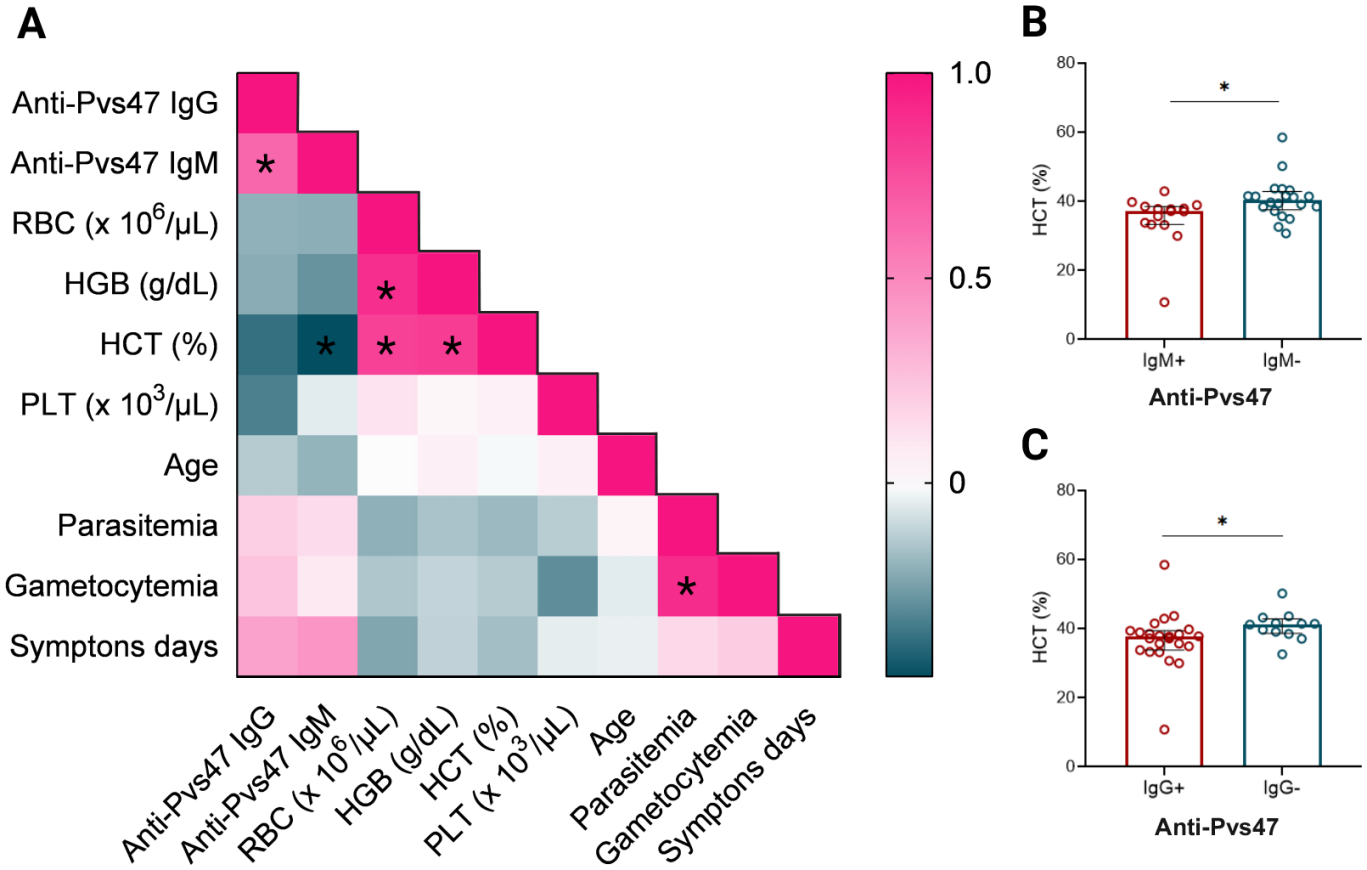
Relationship between hematological parameters and anti-Pvs47 response at Manaus population D0. **(A)** The heatmap represents the correlation between IgM, IgG responses, quantity of erythrocytes (RBC), hemoglobin (HGB), hematocrit percentage (HCT), platelets (PLT), age, parasitemia, gametocytemia and days of symptoms determined by the Spearman test. The color scale indicates the correlation index r; shades of pink represent the most positive correlations (r>0), and shades of turquoise represent the most negative correlations (r<0). **(B)** Comparison between hematocrit levels in anti-Pvs47 IgM reactive (IgM+) and non-reactive individuals (IgM-). **(C)** Comparison between hematocrit levels in anti-Pvs47 IgG reactive (IgG+) and non-reactive individuals (IgG-). Circles indicate the response of each individual. Data from independent experiments are shown as median with interquartile range. The difference between groups was assessed using the Mann−Whitney test, and correlations were evaluated using Spearman’s test. The correlation’s p-value was adjusted using the Holm-Sidak method and deemed significant when p<0.05*.

To assess which factors could be correlated with the anti-Pvs47 response in the Manaus samples, a Spearman correlation analysis was conducted between the IgM and IgG anti-Pvs47 responses, days of symptoms, and hematological parameters (hematocrit, platelet count, and erythrocytes) (**Figure 6A**). A negative correlation was found between the anti-Pvs47 IgM response and hematocrit percentage (HCT) (p=0.033, r= -0.472). Additionally, a positive correlation was observed between anti-Pvs47 IgG and anti-Pvs47 IgM responses (p<0.0005, r= 0.650) Also, a positive correlation was identified with total parasitemia and gametocytemia levels (p<0.005, r=0.895). We compared the hematocrit percentage between anti-Pvs47 IgM reactive and non-reactive patients (**Figure 6B**), as well as anti-Pvs47 IgG reactive and non-reactive patients (**Figure 6C**). In both cases, we observed that patients with higher levels of antibodies had lower hematocrit percentage.

### 3.8 Anti-Pvs47 antibody response is correlated with the blood-stage antigen-specific antibody response

**Figure 7:**
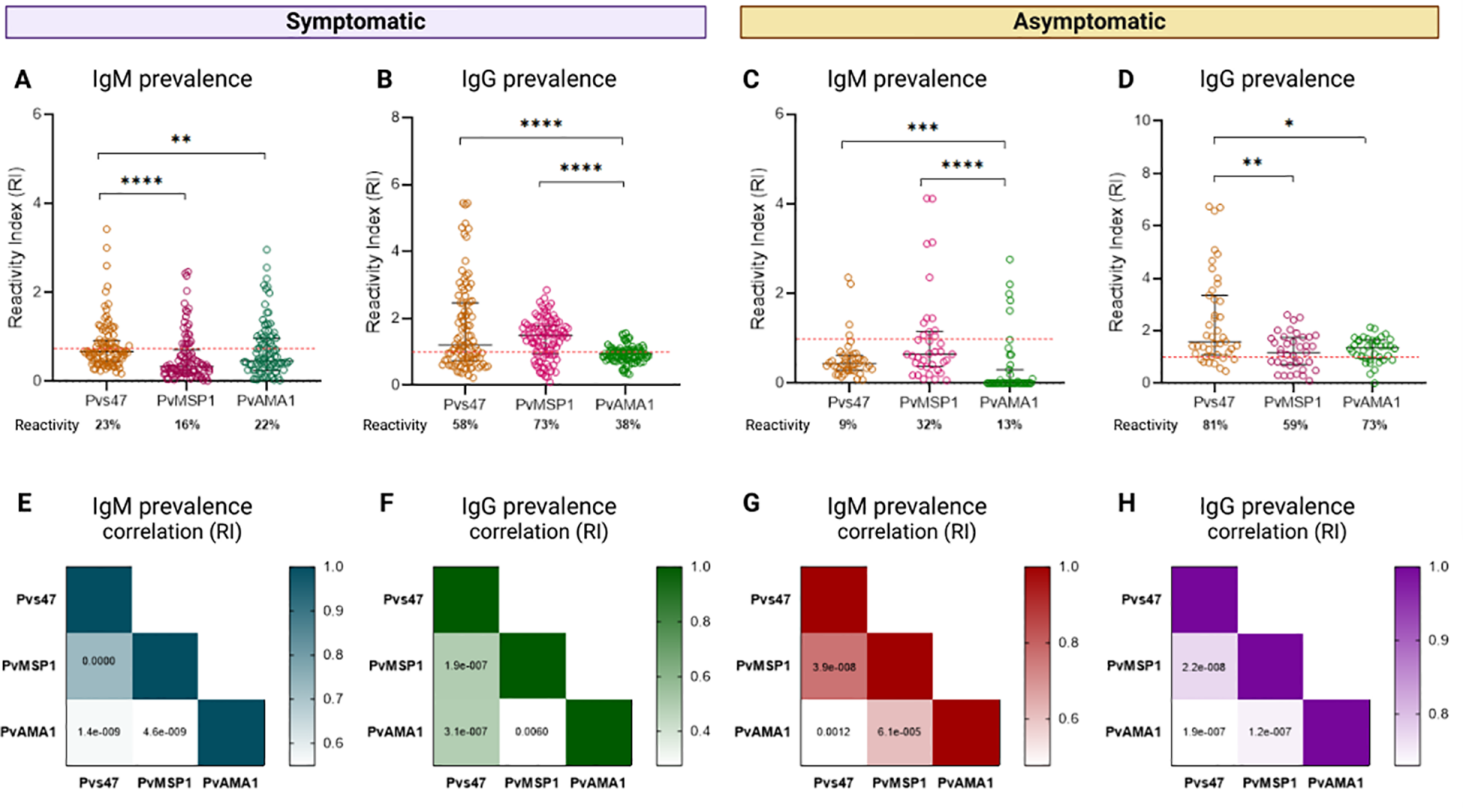
Relationship between the prevalence of anti-Pvs47 antibodies and the response to blood-stage antigens. **(A)** Reactivity index for IgM against Pvs47, PvAMA-1, and PvMSP1 in symptomatic patients from Boa Vista. **(B)** Reactivity index for IgG against Pvs47, PvAMA-1, and PvMSP1 in symptomatic patients from Boa Vista. **(C)** Reactivity index for IgM against Pvs47, PvAMA-1, and PvMSP1 in asymptomatic patients from Presidente Figueiredo. **(D)** Reactivity index for IgG against Pvs47, PvAMA-1, and PvMSP1 in asymptomatic patients from Presidente Figueiredo. **(E)** Correlation of the IgM response across the three antigens in the sample of Boa Vista. **(F)** Correlation of the IgG response across the three antigens in the Boa Vista sample. **(G)** Correlation of the IgM response across the three antigens in the Presidente Figueiredo sample. **(H)** Correlation of the IgG response across the three antigens in the Presidente Figueiredo sample. The RI was calculated from the OD of the individual samples divided by the cutoff. Samples with an RI>1 (indicated by the red dotted line) were considered positive. The circle symbols indicate the response of each individual. The data from independent experiments are shown as median with interquartile range. The color scale in the heatmap indicates the correlation index r of the RI of each antigen response; the darkest color represents the most positive correlation, and the lightest color represents the most negative correlation. A significance level of *p<0.05 was established, and significant p values are depicted in the heatmap. * p < 0,05; ** p < 0,01; ***p < 0,001; ****p < 0,0001.

To elucidate the relationship between the anti-Pvs47 response and the response to the blood stage, we evaluated the antibody response to two others extensively characterized *P. vivax* blood stage antigens, PvAMA1 and PvMSP1_19_, in samples from Boa Vista and Presidente Figueiredo while simultaneously investigating the response to the sexual stage (**Figure 7**, **Supplementary Figure S2**).

Among symptomatic individuals from Boa Vista, the prevalence of IgM and IgG anti-Pvs47 was similar to the responses directed against the classic blood-stage antigens PvAMA1 or PvMSP1_19_. Specifically, IgM reactivity to Pvs47 reached 23%, contrasting with 22% for PvAMA1 and 16% for PvMSP1_19_ (**Figure 7A**). For IgG, 58% showed reactivity to Pvs47, whereas only 38% for PvAMA1 and 73% for PvMSP1_19_, revealing significant differences between the responses to the different antigens (**Figure 7B**).

In the asymptomatic samples of Presidente Figueiredo, we observed a predominant IgM response to the blood-stage antigens, surpassing the response to the sexual-stage antigen, with only 9% reactivity to Pvs47, compared to approximately 13% and 32% for PvAMA1 and PvMSP1_19_, respectively (**Figure 7C**). Regarding the IgG response, we observed a reactivity of 81% to Pvs47, 73% to PvAMA1, and 59% to PvMSP1_19_, indicating significant differences between the groups (**Figure 7D**).

Additionally, we observed a strong positive correlation between the IgM (**Figures 7E, G**) and IgG (**Figures 7F, H**) responses for each antigen in both samples via Spearman correlation analysis. These results demonstrated a significant correlation between the immune response to the blood stage, anti-PvAMA1 and anti-PvMSP1_19_, and the response to the sexual stage, anti-Pvs47, which reflets the correlation of the total parasitemia and gametocytemia levels.

## 4 Discussion

Our study shows that 58% of people, both symptomatic and non-symptomatic for malaria, have antibodies against the Pvs47 protein in their blood. Interestingly, the anti-Pvs47 IgM response appears to be contributing to the clearance of gametocytes in symptomatic patients. These findings are potentially linked to our *in silico* predictions, where we uncovered antigenic and immunogenic properties inherent to Pvs47.

The conformational epitopes predicted here could serve as binding sites for these anti-Pvs47 antibodies. Even more, we identified a specific site as the primary ligand for CD4^+^ cells within the transmembrane region of Pvs47 while CD8^+^ binding peptides are distributed along the sequence. Although these results are i*n silico* prediction, it supports that all the protein could be antigenic. These epitopes hold promise for developing a recombinant subunit vaccine aimed at directing the host immune response toward these immunodominant regions. Besides that, we also revealed a low polymorphism rate (2.5%) among different sequences deposited in databases for Pvs47. These collective results underscore the potential of Pvs47 as a viable vaccine target due to its relative conservation rate and possible capacity to generate antibodies capable of block different *P. vivax* strains. But several experiments should be performed to confirm these predictions.

We have successfully expressed the recombinant Pvs47 in the eukaryotic system of ExpiCHO cells. Our discovery of multiple post-translational modifications, such as glycosylation, disulfide bonds, and phosphorylation sites, suggests that these could impact the epitope structure and its recognition by antibodies. There is evidence highlighting the importance of the phosphorylation process in regulating the parasite’s life cycle, particularly during its sexual stage (49, 50). Therefore, this could also be crucial for the function of Pvs47. Pvs47 protein is largely extracellular, with a few amino acids intracellular, one of which is a serine, a potential phosphorylation site, which could serve as a signal transductor site. These findings indicate that the prokaryotic expression system may not be appropriate for expressing this antigen.

It was once thought that *Plasmodium* lacked N-glycosylation machinery (51). However, recent proteomic data reveals N-glycosylated peptides in key parasite proteins, such as CSP and TRAP (52). N-glycosylation has been detected in the ring, trophozoite, and schizont stages, and is associated with the differentiation of intraerythrocytic stages of the parasite (53–55). We predicted potential N-glycosylation sites in Pvs47. While these predictions suggest possible modifications, the presence of proline (P) at certain positions generally hinders glycosylation (56–58). Thus, further studies are necessary to confirm N-glycosylation in Pvs47. However, the recombinant protein expressed in ExpiCHO cells does exhibit glycosylation sites. Nonetheless, the limited number of these sites is unlikely to significantly alter the protein’s conformation or affect antibodies recognition, as evidenced by the effective recognition of the protein by native antibodies.

The protein was naturally recognized by antibodies from infected individuals, with some showing an immune response lasting up to 50 and 180 days, although this response waned over time. However, the impact of asymptomatic infections in these individuals remains unclear due to limitations in our follow-up protocol. We only assessed parasite presence on days 50 and 180, without considering potential asymptomatic infections that may have occurred in the interval. As a result, the observed duration of antibody responses may be overestimated, suggesting that the true kinetics of antibody persistence after a single infection may be shorter than our data indicate. This limitation highlights the need for further research to more accurately assess the persistence and dynamics of antibody responses.

Previous studies showed the presence of a long-lasting IgM response against blood stage antigens after *P. vivax* infections in areas of low transmission (59). Besides that, in hypoendemic regions, it is suggested that these infections could alter peripheral B-cell subsets, eliciting a persistent parasite-specific IgM response (60). Some studies demonstrate IgM ability to reduce the risk of clinical malaria, including reducing the invasion of red blood cells by merozoites in a complementary system-dependent manner (61). Furthermore, the IgM response appears rapidly in *P. falciparum* infections and is even more pronounced in patients who have had multiple exposures (61). However, little is known about the IgM response to sexual stage antigens. Nevertheless, in the populations samples studied here, although all patients reported previous exposure to the parasite, a low prevalence of IgM antibodies was observed (<37%). However, both symptomatic samples showed superior IgM reactivity compared to the asymptomatic samples. This may be related to the higher level of gametocytes in the circulation in symptomatic patients.

A recent study observed an association between IgM response and *P. vivax* infection in neotropical primates (62). This study suggests that the presence of PvCSP- or PvAMA1-specific IgM could be considered a marker of infection (62). Here we demonstrate that the anti-Pvs47 IgM response is negatively correlated with gametocytemia levels, whereas the anti-MSP1_19_ and anti-AMA1 responses are not. Additionally, higher responders exhibited significantly fewer gametocytes than lower responders. These data support the hypothesis that the anti-Pvs47 IgM response should be crucial for gametocyte elimination, and possible to transmission blocking activity. There is evidence that naturally acquired antibodies against *P. falciparum* gametocytes can affect the morphology and fitness of gametocytes (63). They can even act to reduce gametocytemia, interfere with maturation and reduce the number of oocysts developed in the midgut of mosquitoes, which has already been observed in membrane feeding assays (63). Recently, it was reported that patient-derived purified IgM targeting sexual antigens is significantly more potent than IgG in mediating complement fixation and activation. It was demonstrated that IgM effectively fixed C1q on whole gametocytes and promoted the formation of the C5b-C9 complex on whole gametocytes (64).

Furthermore, we demonstrated that asymptomatic individuals exhibited a higher anti-Pvs47 IgG response, which may be associated with a decrease in gametocyte burden. Specific IgG responses are long-lasting in areas of high transmission. There is evidence that in these regions, where individuals are repeatedly exposed to infection, there is development of more long-lived antibodies compared to areas with low transmission (65). In addition, even in the absence of continuous exposures to the parasite, there are reports of the presence of long-lived antibody responses specific to *P. vivax* (59, 66). It is possible, therefore, that patients who had antibodies over 180 days developed after multiple infection with the parasite.

We also showed that the IgG3 response against Pvs47 had a high prevalence in the three samples studied. The prevalence of IgG3 is considered an important serological marker of *P. vivax* infection (67). Previous studies in *P. falciparum* have shown that IgG3 requires continuous stimulation to maintain effective levels of protection, since IgG3 acts directly and indirectly to fight infection through monocyte activation (68, 69). Nevertheless, the specific response of IgG3 to sexual stages, particularly to Pvs47, remains unclear. It is plausible that IgG3 targeting gametocytes could induce gamete lysis, supported by evidence that sera mediating gamete lysis contain IgG1 and IgG3 specific to gamete surface proteins (70).

Interestingly, asymptomatic patients had high titers of IgG3 and IgG4 subclasses and, to a lesser extent, of IgG1 and IgG2. *P. vivax* infections typically induce an IgG1 and IgG3 subclass response with minimal IgG2 and IgG4 response (59, 71, 72). These subclasses are fundamental components of Fc receptor-mediated responses and are therefore related to phagocytosis responses and antibody-dependent cytotoxicity (ADCC) (73, 74). Evidence suggests that phagocytosis correlates with the presence of IgG1 antibodies against sexual stage antigens (75). This suggests that the functional efficacy of naturally acquired anti-Pvs47 antibodies could be augmented by complement activation, although additional investigation is warranted.

In non-immune individuals who have been previously exposed to the pathogen but have not developed an efficient immune response, there is a predominance of IgG2 and IgG4 responses (76). In addition, the IgG2 and IgG4 response have been associated with cerebral malaria (77, 78). Interestingly, the presence of IgG4 antibodies against Pvs47 was identified in the samples studied here (21% in Presidente Figueiredo, 27% in Manaus and 6.5% in Boa Vista). The IgG4 response is less frequent and less reported in *Plasmodium* infections. There are reports of associations of IgG4 with a high risk of infection, and some studies suggest that the IgG4 response could block the effect of cytophilic antibodies (74, 79). In addition, the presence of IgG4 antibodies specific to the salivary proteins of the anopheline mosquito has already been described in the literature (80). In fact, it is suggested that this response is related to an allergic response and immune tolerance due to repeated exposure to mosquito saliva (80). For Pvs47, the prevalence of IgG4 response was observed in the three populations samples. This response may be related to high susceptibility to repeated *Plasmodium* infections (79). However, understanding the dynamics of the IgG4 response remains elusive for most antigens, particularly those associated with the sexual stage.

We also found a negative correlation of anti-Pvs47 response with hematocrit levels in Manaus samples. Similarly, a recent study identified a negative correlation between the anti-Pvs230 antibody response and hemoglobin concentration (72). Possibly, this correlation is due to anemia and thrombocytopenia developed in patients at the beginning of the infection due to the presence of the parasite in the circulation (81). It is suspected that adhesive phenotypes contribute to anemia, either in the sequestration of the parasite to the tissues or in the formation of rosettes (9). Studies report the ability of *P. vivax* gametocytes to form rosettes, which may impact mosquito infectivity (21, 22). In fact, in *P. falciparum*, the prevalence of gametocytes in the bone marrow has been associated with severe anemia and dyserythropoiesis (82). In addition, some studies suggest that bone marrow provides an environment conducive to asexual replication and gametocyte development (83). These observations suggest that the anti-Pvs47 response may influence hematological parameters, however, further research is needed to better understand the specific mechanisms underlying this association.

Our study unveiled a positive correlation between the anti-Pvs47 response and the response to blood stages, particularly regarding PvMSP1_19_ and PvAMA1. This could reflect the positive correlation between total parasitemia and gametocytemia (47). Importantly, Pvs47 exhibited higher recognition than conventional antigens among asymptomatic samples, underscoring its significance as a vaccine candidate.

Previous studies indicate that individuals who show a targeted response to Pvs47 have a higher ability to block transmission in membrane feeding assays (27). In this case, a 34% reduction in the infection rate of mosquitoes was observed (27). However, the naturally acquired antibody response was not characterized in this study (27). According to previous studies, the presence of antibodies targeting proteins of the six-cysteine protein family and gametocyte proteins of *P. falciparum* were associated with the ability to block transmission (84). Therefore, there is a strong indication that these antibodies may somehow act to block transmission. We suggest anti-Pvs47 IgM as a key factor in this process, which should be investigated in the future.

In this study, we investigated the naturally acquired immune response to Pvs47, examining the presence of antibodies rather than their functional activity. However, it is suggested that these antibodies may contribute to gametocyte depuration through mechanisms such as complement activation and antibody-dependent cytotoxicity. Besides human studies, it is essential to assess the role of these antibodies in anopheline mosquitoes. Previous studies have already demonstrated the ability to block the transmission of anti-Pvs47 antibodies (27), but the mechanism of action and the ability to interfere with gamete fecundity or sexual stage development remains elusive. Overall, Pvs47 is a potential transmission-blocking vaccine candidate that should be further explored.

## 5 Conclusion

In summary, we showed through *in silico* and serological analysis that the Pvs47 protein is highly antigenic, promoting a naturally acquired response in both symptomatic and asymptomatic individuals. Furthermore, some individuals showed positive responses on day 180. The prevalence of anti-Pvs47 IgG3 subclass antibodies among the three samples suggests the predominance of cytophilic responses to this antigen. Moreover, the anti-Pvs47 IgM seems to be important to control gametocytes development and the negative correlation with hematological parameters suggest a potential effect in hematological niches, which need further examination. The correlation observed between antibody responses to blood stage and sexual stage antigens illustrates the interconnectedness and complexity of the malaria response. These findings are significant for vaccine development, as they suggest the likelihood of natural immune recognition of Pvs47 before vaccination, emphasizing the need for additional investigations to determine whether these acquired antibodies can effectively block transmission. Furthermore, exploring the immunogenicity of Pvs47 in a vaccine formulation is crucial for advancing our understanding of its potential application in malaria prevention strategies.

## Data Availability

The original contributions presented in the study are included in the article/**Supplementary Material**. Further inquiries can be directed to the corresponding author.
