# Supplementary material for: Exploring the naturally acquired response to Pvs47 gametocyte antigen
Source: Front Immunol. 2024 Oct 10;15:1455454. doi: 10.3389/fimmu.2024.1455454 (PMC11499161; doi:10.3389/fimmu.2024.1455454)
Supplement: Supplementary file 1 [file DataSheet1.docx]

Supplementary Material

EXPLORING THE NATURALLY ACQUIRED RESPONSE TO Pvs47 GAMETOCYTE ANTIGEN

Gisele Tatiane Soares da Veiga^1^, Rafael Amaral Donassolo^1^, Sofia Forcellini^2^, Julia Weber Ferraboli^1^, Mario Antonio Kujbida Junior^1^, Líndice Mitie Nisimura^2^, Letícia Werzel Bassai^3^, Rafael Luis Kessler^3^, Mariana Serpeloni^3^, Najara Carneiro Bittencourt^4^, Yanka Evellyn Alves R. Salazar^5,6^, Luiz Felipe Ferreira Guimarães^5^, Jaime Louzada^7^, Dayanne Kamylla Alves da Silva Barros^8^, Stefanie Costa Pinto Lopes^8,9^, Luzia Helena Carvalho^5^, Tais Nóbrega de Sousa^5,6^, Flora Satiko Kano^5^, Fabio Trindade Maranhão Costa^4^, Pryscilla Fanini Wowk^2^, Letusa Albrecht^1*^

*** Correspondence:** letusa.albrecht@fiocruz.br

# Supplementary Figures


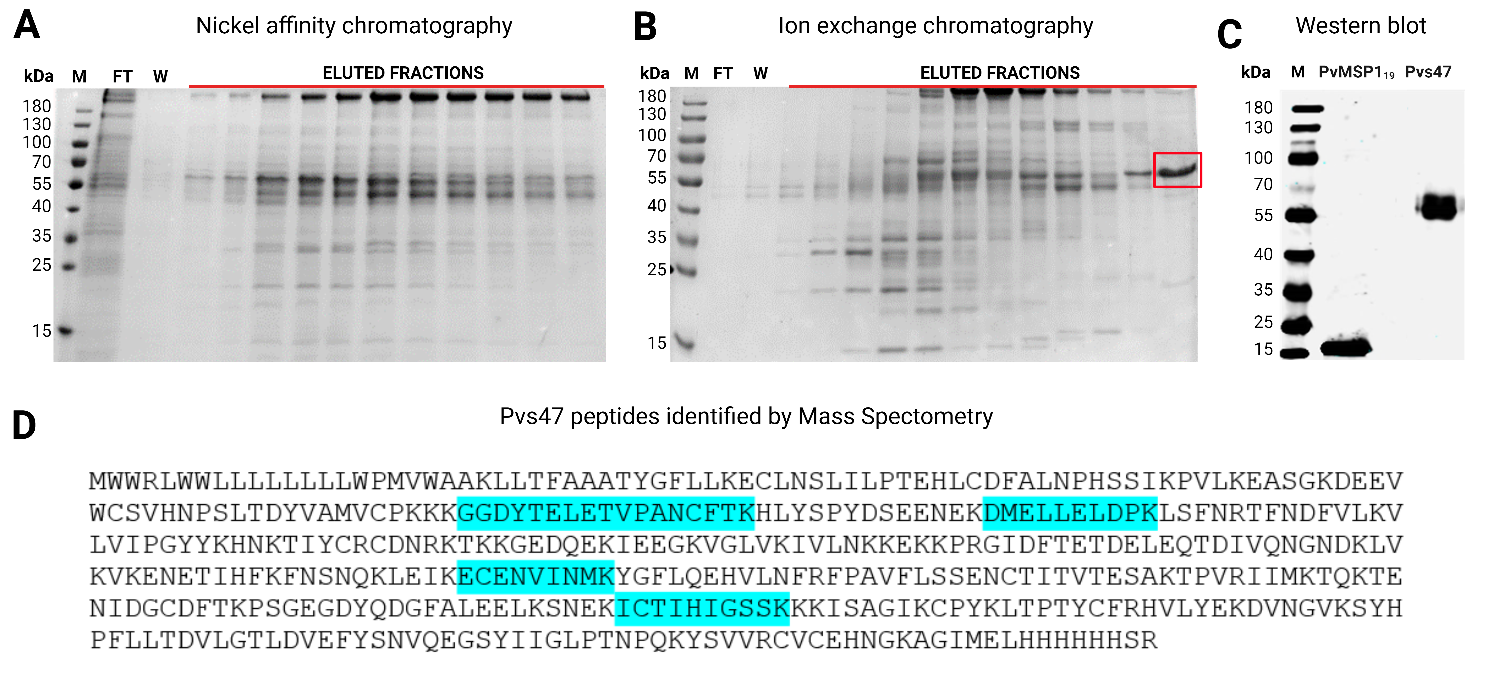


**Figure S1. Confirmation of expression and purification of the protein Pvs47.** A) SDS-PAGE of eluted fractions from nickel affinity chromatography. B) SDS-PAGE of eluted fractions from ion exchange chromatography, highlighting the fraction used in experiments. C) Western blot with anti-histidine antibody identifying the proteins PvMSP1_19_ and Pvs47. D) Peptides from the purified Pvs47 protein identified by mass spectrometry. M- molecular marker, FT- unbound fractions, W- nickel resin wash.


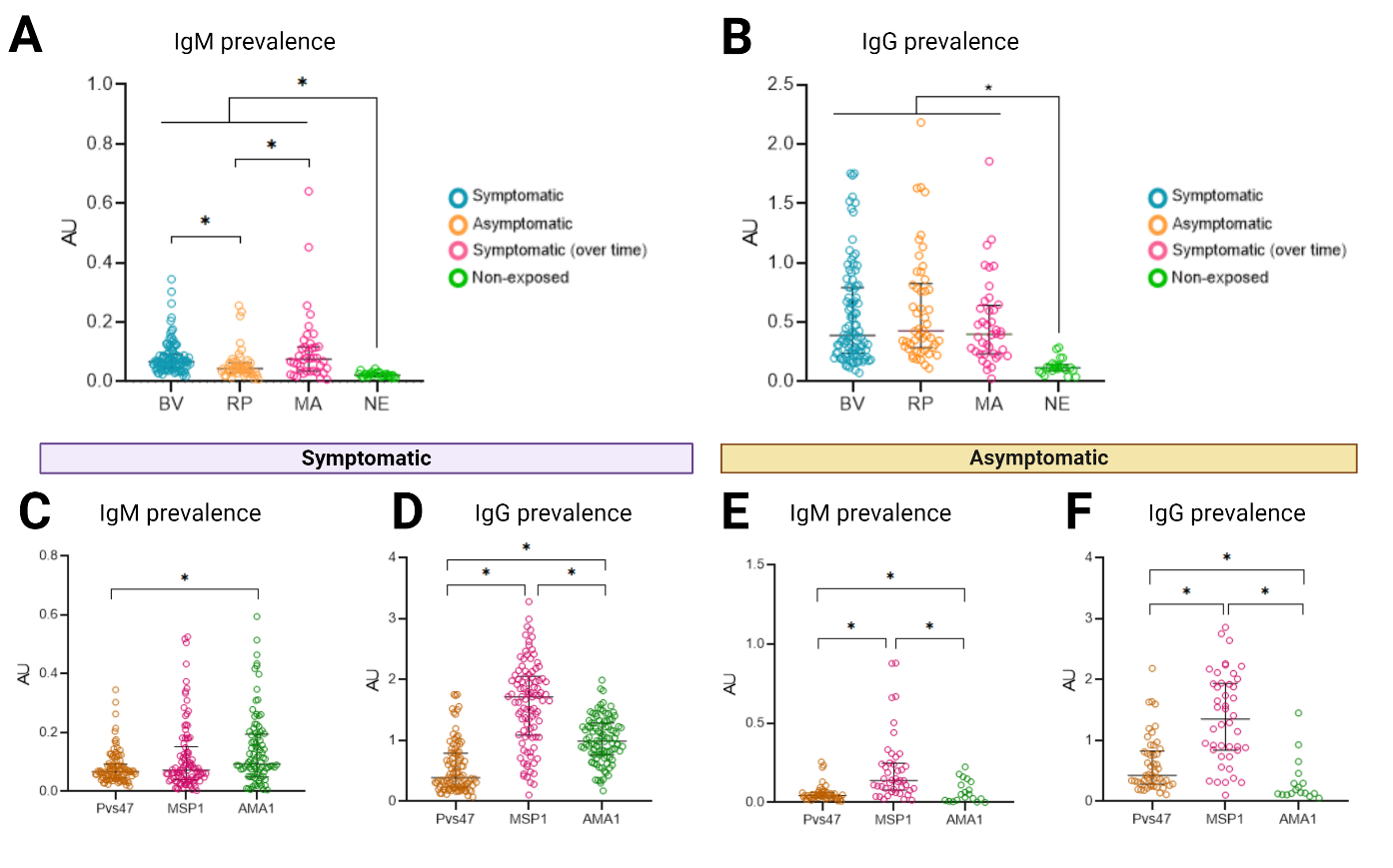


**Figure S2. Anti-Pvs47 antibody response in arbitrary units.** A) IgM anti-Pvs47 response in Boa Vista (BV), Rio Pardo (RP), Manaus (MA), and unexposed individuals (NE). B) IgG anti-Pvs47 response in Boa Vista (BV), Rio Pardo (RP), Manaus (MA), and unexposed individuals (NE). C) IgM response to Pvs47, MSP1, and AMA1 in samples from Boa Vista. D) IgG response to Pvs47, MSP1, and AMA1 in samples from Boa Vista. E) IgM response to Pvs47, MSP1, and AMA1 in samples from Presidente Figueiredo (Rio Pardo). F) IgG response to Pvs47, MSP1, and AMA1 in samples from Presidente Figueiredo (Rio Pardo). Data are represented as the mean optical density for each sample, adjusted by subtraction of the background value, with an additional subtraction of the GST value for AMA1. Each circle represents the response of one individual. Data from independent experiments are shown as medians with interquartile range. p-values ​​were determined using the Kruskal-Wallis test followed by Dunnett's multiple comparisons test (*p < 0.05).


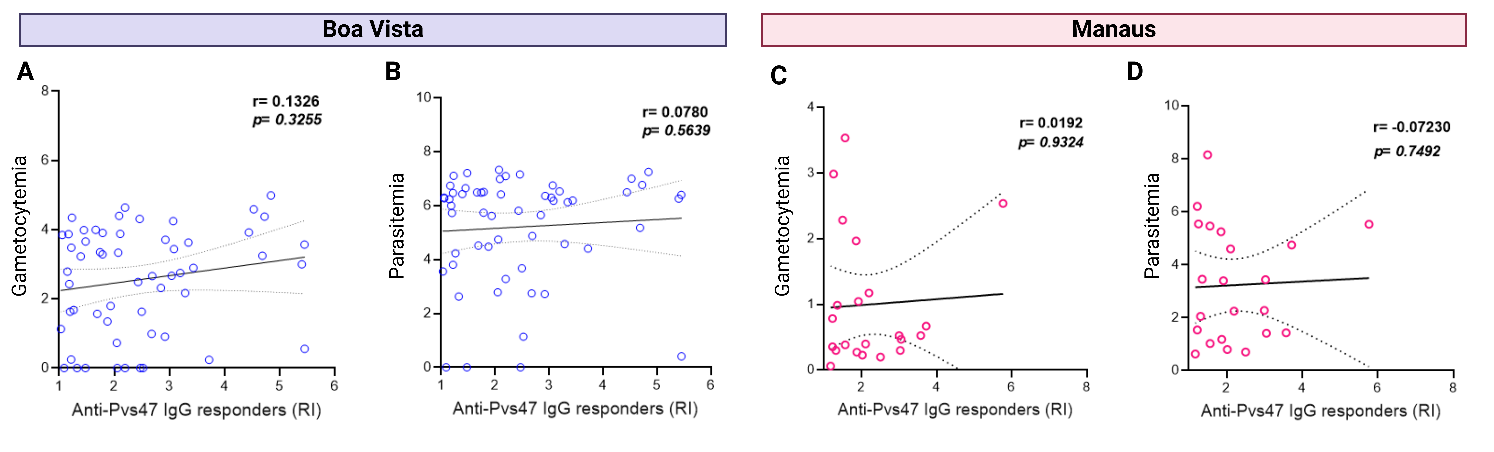


**Figure S3.** **Correlation between** **anti-Pvs47 IgG antibody response and gametocytemia or parasitemia levels.** A) Correlation between the reactivity index (RI) of the anti-Pvs47 IgG response and the levels of gametocytemia in samples from Boa Vista. B) Correlation between the reactivity index (RI) of the anti-Pvs47 IgG response and the levels of parasitemia in samples from Boa Vista. C) Correlation between the reactivity index (RI) of the anti-Pvs47 IgG response and the levels of gametocytemia in samples from Manaus. D) Correlation between the reactivity index (RI) of the anti-Pvs47 IgG response and the levels of parasitemia in samples from Manaus. Circles indicate the response of each individual. The correlation was evaluated by the Spearman’s test and the linear regression line of the data with a 95% confidence interval is represented.


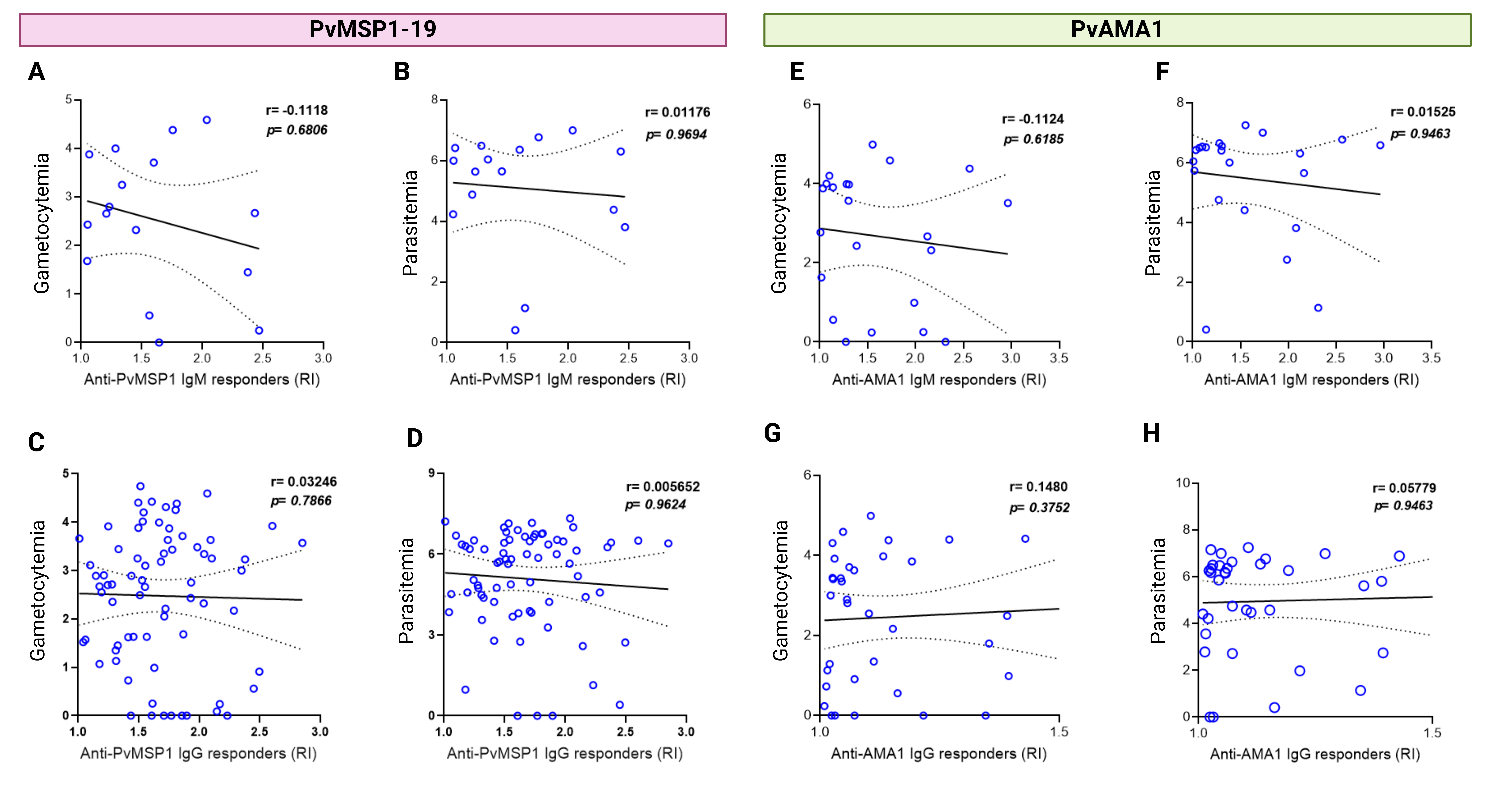


**Figure S4. Relationship between Anti-MSP1 and Anti-AMA1 antibodies response and gametocytemia or parasitemia levels.** A) Correlation between the reactivity index (RI) of the anti-PvMSP1_19_ IgM response and the levels of gametocytemia in samples from Boa Vista. B) Correlation between the reactivity index (RI) of the PvMSP1_19_ IgM response and the levels of parasitemia in samples from Boa Vista. C) Correlation between the reactivity index (RI) of the anti-PvMSP1_19_ IgG response and the levels of gametocytemia in samples from Boa Vista. D) Correlation between the reactivity index (RI) of the PvMSP1_19_ IgG response and the levels of parasitemia in samples from Boa Vista. E) Correlation between the reactivity index (RI) of the anti-PvAMA1 IgM response and the levels of gametocytemia in samples from Boa Vista. F) Correlation between the reactivity index (RI) of the PvAMA1 IgM response and the levels of parasitemia in samples from Boa Vista. G) Correlation between the reactivity index (RI) of the anti-PvAMA1 IgG response and the levels of gametocytemia in samples from Boa Vista. H) Correlation between the reactivity index (RI) of the PvAMA1 IgG response and the levels of parasitemia in samples from Boa Vista. Circles indicate the response of each individual. The correlation was evaluated by the Spearman’s test and the linear regression line of the data with a 95% confidence interval is represented.

# Supplementary Tables

**Table S1. Predicted amino acid composition for Pvs47**

| **Amino acid (AA)** | **AA signal** | **AA number** | **AA Percentage** |
| --- | --- | --- | --- |
| Ala | (A) | 16 | 3.7% |
| Arg | (R) | 9 | 2.1% |
| Asn | (N) | 25 | 5.8% |
| Asp | (D) | 21 | 4.8% |
| Cys | (C) | 16 | 3.7% |
| Gln | (Q) | 9 | 2.1% |
| Glu | (E) | 38 | 8.8% |
| Gly | (G) | 24 | 5.5% |
| His | (H) | 11 | 2.5% |
| Ile | (I) | 25 | 5.8% |
| Leu | (L) | 46 | 10.6% |
| Lys | (K) | 46 | 10.6% |
| Met | (M) | 6 | 1.4% |
| Phe | (F) | 20 | 4.6% |
| Pro | (P) | 18 | 4.2% |
| Ser | (S) | 27 | 6.2% |
| Thr | (T) | 28 | 6.5% |
| Trp | (W) | 2 | 0.5% |
| Tyr | (Y) | 17 | 3.9% |
| Val | (V) | 29 | 6.7% |

**Table S2. Linear B-cell epitopes of the Pvs47 antigen.**

| **Linear B-cell epitopes** | **Position** | **Score** |
| --- | --- | --- |
| SVHNPSLTDYVAMVC | 57 | 0.90 |
| DVLGTLDVEFYSNVQE | 360 | 0.90 |
| KKKISAGIKCPYKLTP | 319 | 0.90 |
| TKHLYSPYDSEENEKD | 91 | 0.88 |
| KDEEVWCSVHNPSLTD | 50 | 0.88 |
| KTENIDGCDFTKPSGE | 277 | 0.888 |
| PRGIDFTETDELEQTD | 179 | 0.88 |
| HVLYEKDVNGVKSYHP | 340 | 0.87 |
| KVKENETIHFKFNSNQ | 205 | 0.87 |
| KHNKTIYCRCDNRKTK | 137 | 0.87 |

*These epitopes were predicted by the ABCpred tool. Red color represents mutations sites of the epitopes. Peptides with score next to 1 represent high binding affinity.

Table S3. MHC-I binding epitopes of the Pvs47 antigen.

| **MHC-I epitopes** | **Position** | **Score** | **Percentile rank** |
| --- | --- | --- | --- |
| RTFNDFVLK | 120 | 0.98 | 0.01 |
| CPYKLTPTY | 328 | 0.98 | 0.01 |
| KENETIHFKF | 207 | 0.97 | 0.01 |
| ALNPHSSIK | 33 | 0.96 | 0.01 |
| SLTDYVAMV | 62 | 0.94 | 0.02 |
| QEHVLNFRF | 239 | 0.94 | 0.02 |
| ETVPANCFTK | 83 | 0.94 | 0.04 |
| LELDPKLSF | 110 | 0.92 | 0.03 |
| ASGKDEEVW | 47 | 0.91 | 0.05 |
| HPFLLTDVL | 354 | 0.89 | 0.04 |
| GLVKIVLNK | 166 | 0.89 | 0.03 |
| VLYEKDVNGV | 341 | 0.87 | 0.03 |
| ECENVINMKY | 226 | 0.86 | 0.05 |
| SVHNPSLTDY | 57 | 0.86 | 0.03 |
| YSNVQEGSY | 370 | 0.86 | 0.05 |

*MHC-I binding epitopes were predicted by the MHCI IEDB tool. Red color represents mutations sites of the epitopes. Peptides with score next to 1 and low percentile rank represent high binding affinity.

Table S4. MHC-II binding epitopes of Pvs47.

| **MHC-II epitopes** | **Position** | **Percentile rank** |
| --- | --- | --- |
| AFLSLTLLLLLIAL | 416 | 0.01 |
| ASSSGWAFLSLTLLLL | 410 | 0.01 |
| FLSLTLLLLLIALLS | 417 | 0.01 |
| GWAFLSLTLLLLLI | 414 | 0.01 |
| SGWAFLSLTLLLLL | 413 | 0.01 |
| SSGWAFLSLTLLLL | 412 | 0.01 |
| SSSGWAFLSLTLLLL | 411 | 0.01 |
| WAFLSLTLLLLLIA | 415 | 0.01 |
| IASSSGWAFLSLTLLLLL | 409 | 0.01 |
| RIASSSGWAFLSLTLLLL | 408 | 0.03 |
| LSLTLLLLLIALLSAC | 418 | 0.05 |
| LTLLLLLIALLSA | 420 | 0.09 |
| SLTLLLLLIALLS | 419 | 0.09 |
| TLLLLLIALLSAC | 421 | 0.09 |
| IHFKFNSNQKLEIK | 212 | 0.11 |
| TIHFKFNSNQKLEI | 211 | 0.11 |
| ELLELDPKLSFNR | 108 | 0.12 |
| MELLELDPKLSFN | 107 | 0.13 |
| ETIHFKFNSNQKLEIK | 210 | 0.13 |
| DMELLELDPKLSFN | 106 | 0.15 |
| KDMELLELDPKLSFN | 105 | 0.17 |
| NETIHFKFNSNQKLEIK | 209 | 0.19 |

*The MHC-II binding epitopes were predicted by the NetMHCpan EL 4.1 predictor. Peptides with low percentile rank represent high binding affinity.

**Table S5. The nonlinear epitopes from the Pvs47 antigen generated from the 3D modeled structure.**

|  | **Epitopes** | | **Residues** | **Score** |
| --- | --- | --- | --- | --- |
| **1** | | A:R180, A:G181, A:I182, A:D183, A:F184, A:T185, A:E186, A:T187, A:D188, A:E189, A:L190, A:E191, A:Q192, A:T193, A:D194, A:I195, A:V196, A:Q197, A:N198, A:G199, A:N200, A:D201, A:K202, A:L203, A:V204, A:K205, A:K207, A:F214, A:K215, A:F216, A:N217, A:S218, A:N219, A:Q220, A:K221, A:L222, A:E223, A:I224, A:K225, A:E226, A:C227, A:E228, A:N229, A:V230, A:H241, A:F251, A:L252, A:S253, A:S254, A:E255, A:N256, A:C257, A:T258, A:I259, A:T260, A:V261, A:T262, A:E263, A:S264, A:A265, A:K266, A:T267, A:P268, A:V269, A:R270, A:I271, A:I272, A:K274, A:Q276 | 69 | 0.734 |
| **2** | | A:C284, A:D285, A:F286, A:T287, A:K288, A:P289, A:S290, A:G291, A:E292, A:G293, A:D294, A:Y295, A:D297, A:G298, A:F299, A:A300, A:L301, A:E302, A:E303, A:L304, A:K305, A:S306, A:N307, A:E308, A:K309, A:I310, A:C311, A:T312, A:H314, A:I326, A:K327, A:C328, A:P329, A:Y330, A:K331, A:L332, A:T333, A:P334, A:N372, A:V373, A:Q374, A:E375, A:G376, A:S377, A:Y378, A:V395, A:C396, A:E397, A:H398, A:N399, A:G400, A:K401, A:A402, A:G403, A:I404, A:E406, A:R408 | 57 | 0.723 |
| **3** | | A:A33, A:L34, A:S39, A:I40, A:V43, A:L44, A:E46, A:A47, A:S48, A:G49, A:K50, A:D51, A:E52, A:E53, A:C71, A:P72, A:K73, A:K74, A:K75, A:G76, A:G77, A:D78, A:Y79, A:T80, A:E81, A:L82, A:E83, A:T84, A:V85, A:P86, A:A87, A:N88, A:C89, A:T91, A:K92, A:L94, A:Y95, A:S96, A:P97, A:Y98, A:D99, A:S100, A:E101, A:E102, A:N103, A:E104, A:K105, A:E108, A:L109, A:L110, A:E111, A:F118, A:N119, A:R120, A:T121, A:F122, A:N123, A:D124, A:F125, A:V126, A:Y136, A:K137, A:H138, A:N139, A:R145, A:C146, A:D147, A:N148, A:R149, A:K150, A:T151, A:K152, A:K153, A:G154, A:E155, A:D156, A:Q157, A:E158, A:K159, A:I160, A:E161, A:E162, A:G163, A:K164, A:V165, A:G166 | 86 | 0.686 |
| **4** | | A:S317, A:S318, A:K319, A:A410, A:S411, A:S412, A:S413, A:G414, A:W415, A:A416, A:F417, A:L418, A:S419, A:L420, A:T421, A:L422, A:L423, A:L424, A:L425, A:L426, A:I427, A:A428, A:L429, A:L430, A:S431, A:A432, A:C433 | 27 | 0.595 |
| **5** | | A:K345, A:D346, A:V347, A:N348 | 4 | 0.505 |

*Red color represents mutations sites of the epitopes. Score >0.5 are predicted as positive.
